# Conjugation of Doxorubicin to siRNA Through Disulfide-based Self-immolative Linkers

**DOI:** 10.3390/molecules25112714

**Published:** 2020-06-11

**Authors:** Florian Gauthier, Jean-Rémi Bertrand, Jean-Jacques Vasseur, Christelle Dupouy, Françoise Debart

**Affiliations:** 1 IBMM, University of Montpellier, CNRS, ENSCM, 34095 Montpellier, France; flo.gauthier.43@gmail.com (F.G.); francoise.debart@umontpellier.fr (F.D.); 2 METSY UMR 9018 CNRS, Université Paris-Sud, Gustave Roussy, University Paris-Saclay, 94800 Villejuif Cedex, France; Jean-remi.BERTRAND@gustaveroussy.fr

**Keywords:** doxorubicin, siRNA, conjugation, disulfide bond, self-immolative linker, thiol-disulfide exchange

## Abstract

Co-delivery systems of siRNA and chemotherapeutic drugs have been developed as an attractive strategy to optimize the efficacy of chemotherapy towards cancer cells with multidrug resistance. In these typical systems, siRNAs are usually associated to drugs within a carrier but without covalent interactions with the risk of a premature release and degradation of the drugs inside the cells. To address this issue, we propose a covalent approach to co-deliver a siRNA-drug conjugate with a redox-responsive self-immolative linker prone to intracellular glutathione-mediated disulfide cleavage. Herein, we report the use of two disulfide bonds connected by a pentane spacer or a *p*-xylene spacer as self-immolative linker between the primary amine of the anticancer drug doxorubicin (Dox) and the 2′-position of one or two ribonucleotides in RNA. Five Dox-RNA conjugates were successfully synthesized using two successive thiol-disulfide exchange reactions. The Dox-RNA conjugates were annealed with their complementary strands and the duplexes were shown to form an A-helix sufficiently stable under physiological conditions. The enzymatic stability of Dox-siRNAs in human serum was enhanced compared to the unmodified siRNA, especially when two Dox are attached to siRNA. The release of native Dox and RNA from the bioconjugate was demonstrated under reducing conditions suggesting efficient linker disintegration. These results demonstrate the feasibility of making siRNA-drug conjugates via disulfide-based self-immolative linkers for potential therapeutic applications.

## 1. Introduction

Successful chemotherapy of cancer using small-molecule drugs has to overcome a major obstacle that is the multidrug resistance of cancer cells [1]. Since several decades, great research efforts have been provided to circumvent the resistance and consequently to improve chemotherapy. In this aim, combination therapy of nucleic acids and anticancer drugs was proposed to increase therapeutic efficacy and prevent side effects [2,3].

The anthracyclines and more specifically doxorubicin (Dox) are efficient anticancer molecules acting as DNA intercalating agents and DNA topoisomerase II inhibitors for the treatment of leukemia, lymphoma or breast cancer [4,5]. However, despite Dox efficacy, high doses of Dox are toxic for organs and particularly result in heart damage [6]. Therefore, the association of several therapeutic agents with different cellular targets was proposed to decrease their dose and consequently their toxicity. In particular, the dual delivery of anticancer drugs as apoptosis inducers and small interfering RNAs (siRNAs) [7,8] as oncogene silencers was envisaged to enhance synergistic effects. This combination is expected to increase the cellular uptake and efficiency while reducing the amount of drugs used and consequently offsetting their side effects [3,9,10,11,12].

Numerous examples of co-delivery systems of anticancer drugs and siRNA using nanoparticles as carriers have been described [13,14,15]. For example, copolymer micelles encapsulating both Dox by hydrophobic interactions and siRNA by electrostatic interactions were more efficient on a breast cancer cell line than a sequential administration of two separate drugs [11,16]. Inorganic nanoparticles such as mesoporous silica nanoparticles have been developed for the targeted simultaneous delivery of Dox and siRNA into breast cancer cells [17,18,19]. Selenium nanoparticles have received an increasing attention as drug carriers for the targeted co-delivery of Dox and siRNA in vitro and in vivo [20]. All these co-delivery systems with non-covalent interactions between anticancer drugs and siRNA exhibit a major drawback that might be a premature release and a lysosomal degradation before the release of the drugs inside the cells. This disadvantage may be circumvented by a covalent approach to co-deliver the conjugate siRNA-drug. So far, to our knowledge, no molecular bioconjugate using a covalent linker between Dox and siRNA has been reported.

The covalent co-delivery strategy implies the recovery of both separate active drugs in response to a cellular stimulus. Therefore, as a conventional linker between siRNA and drug can modify their intrinsic properties, self-immolative linkers (SILs) are assets to achieve this objective [21]. They provide a robust strategy to bring both drugs simultaneously inside cells until external stimuli trigger the SIL disintegration via fragmentation or intramolecular cyclization processes, releasing the native functional biomolecules [22]. Disulfide bonds provide biological relevant precursors to initiate self-immolation in the presence of reducing agents such as glutathione (GSH) [23]. Redox-responsive disulfide linkages take advantage of extracellular and intracellular GSH gradients. Indeed, disulfide-based linkers confer high stability to the delivery system outside cells (GSH concentration 2–10 µM in body fluids) whereas they are rapidly cleaved within the cytosol (GSH concentration 1–10 mM) [24]. More importantly, these redox-responsive linkers are particularly relevant in cancer therapy [25], since many tumor and cancer cells were found to contain elevated GSH levels in comparison with normal tissues and cells [26]. Furthermore, it is worth mentioning that disulfide bridge has been widely used as a linker in nucleic acids bioconjugation as thoroughly described in two recent 2-part article reviews [27,28].

**Scheme 1:**
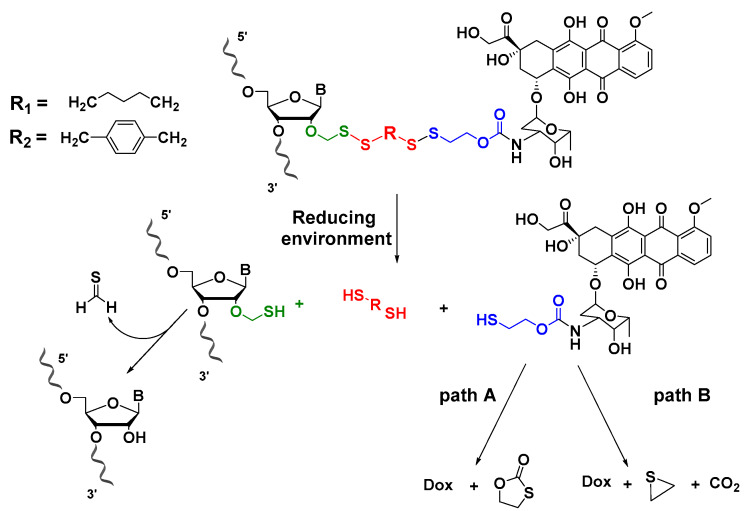
Proposed mechanism for the self-immolation of Dox-RNA conjugates under reducing conditions.

The objective of this work was to conjugate Dox to the sense strand of a siRNA targeting the production of the EWS/Fli-1 protein involved in Ewing’s sarcoma [29] through a disulfide-based self-immolative linker. Here we describe the post-synthesis conjugation of one or two Dox to RNA using two different linkers. Each linker was designed with two disulfide bridges separated by an aliphatic pentane spacer (R_1_) or an aromatic *para*-xylene spacer (R_2_). With this aim, the amino function of Dox was linked to one disulfide bridge through an ethyl carbamate bond and the 2′-*O*-position of ribonucleotides in RNA was connected to the other disulfide bond through a methylene group (Scheme 1).

The synthesis of Dox-RNA conjugates is partly based on a thiol-disulfide exchange reaction previously described by our group for the conjugation of RNA with various biomolecules (anticancer drugs, peptides, lipids, carbohydrates or fluorescence probes) [30] and further exploited in a siRNA prodrug approach [31]. The SILs between Dox and RNA were designed so that the reduction of the disulfide bonds releases both unstable 2’-hemithioacetal RNA and 2-thioethylcarbamate Dox. Then, on one hand, the hemithioacetal evolves spontaneously towards the recovery of 2′-OH RNA by loss of thioformaldehyde as shown previously [31,32]. On the other hand, the release of native doxorubicin takes place through a rearrangement according to two mechanisms [24,33] with either elimination of 1,3-oxathiolan-2-one (Path A) [34], or of ethylene sulfide and carbon dioxide (Path B) [35,36].

To assess the impact of the Dox positioning in a 21-mer RNA sequence (EWS/Fli-1 sense strand 5′-GCA GCA GAA CCC UUC UUA UGA-3′) on the biophysical properties of several Dox-siRNA conjugates, one Dox was attached either at 2′-position of guanosine G_1_ at 5′-end of the RNA sequence or at 2′-position of cytidine C_11_ in the middle of the sequence. Furthermore, two Dox molecules were attached to both locations (G_1_ and C_11_) in the sequence. The biophysical properties of Dox-siRNA conjugates were studied by UV thermal denaturation and CD spectroscopy. The enzymatic stability of Dox-RNA conjugates was evaluated in human serum. Finally, the effective cleavage of the disulfide bonds by a reducing agent followed by the self-immolation of the linkers, and the resulting release of both native drugs were demonstrated.

## 2. Results and Discussion

### 2.1. Synthesis of Dox-RNA Conjugates

**Scheme 2:**
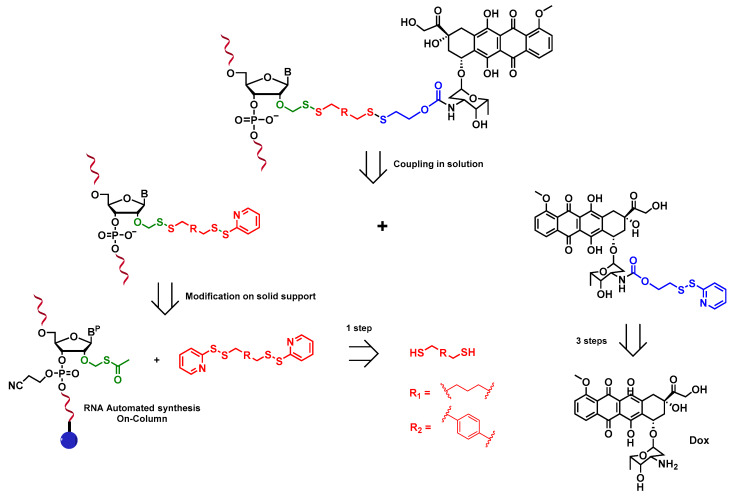
Retrosynthetic analysis for Dox-RNA conjugates.

Dox-RNA conjugates have been synthesized in two steps following the retrosynthetic analysis depicted in Scheme 2. The first step was the separate preparation of both partners of the conjugates. Dox was functionalized at the amino function by a 2-(disulfanylpyridine)-ethylcarbonate moiety according a 3-steps published procedure [37]. 2′-*O*-activated RNAs were synthesized on-column by reaction of an alkylbis(disulfanylpyridine) reagent on a 2′-*O*-thioacetyl ribonucleoside introduced into the RNA sequences. The second step was the final coupling in solution of modified Dox with 2′-*O*-activated RNAs to give the conjugates.

#### 2.1.1. Synthesis of Alkylbis(disulfanylpyridine) Reagents 1 and 2

**Scheme 3:**
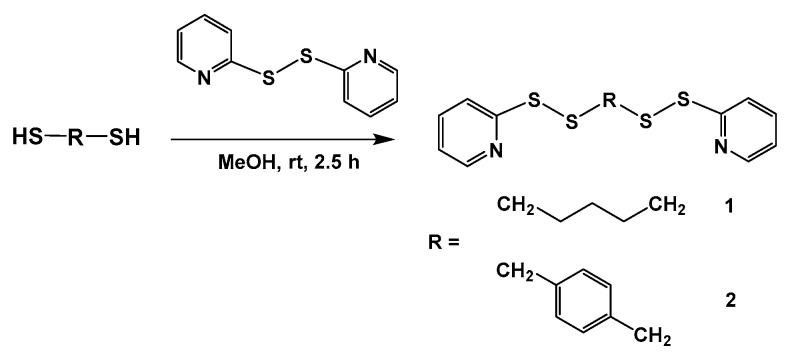
Synthesis of 1,5-bis(pyridin-2-yldisulfanyl)-pentane **1** and bis(pyridin-2-yldisulfanyl)-*p*-xylene **2.**

Two different spacer precursors containing two disulfide bonds linked through an aliphatic pentyl chain (R**_1_**) or a *p*-xylenyl moiety (R_2_) were prepared to functionalize RNA. The reaction was carried out in dry methanol for 2.5 h with commercial pentane-1,5-dithiol or 1,4-benzenedimethanethiol in the presence of 2,2′-dithiodipyridine, affording 1,5-bis(pyridin-2-yldisulfanyl)-pentane **1** (31%) and bis(pyridin-2-yldisulfanyl) *p*-xylene **2** (37%), respectively (Scheme 3). Possible polymerization of the dithiols reagents might explain these moderate yields.

#### 2.1.2. Synthesis of 2′-O-activated RNAs

The reagents **1** and **2** were used to modify a 21-mer RNA, corresponding to the sense strand S of siEF-4, siRNA targeting the gene coding for the fusion protein EWS/Fli1, involved in Ewing’s sarcoma [29,38]. The linkers were attached to G_1_ nucleoside at the 5′end of the sequence (G_1_-R_1_ and G_1_-R_2_) or to C_11_ nucleoside in the middle of the sequence (C_11_-R_1_ and C_11_-R_2_) or to both positions (G_1_C_11_-R_2_; Table 1).

**Scheme 4:**
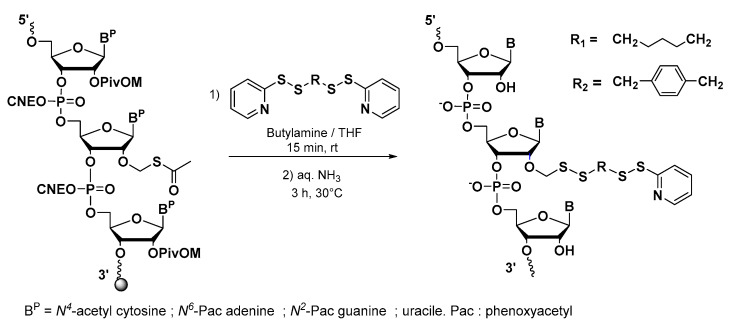
Synthesis of 2′-*O*-PySS**R_1_**SSMe and 2′-*O*-PySS**R_2_**SSMe RNAs.

RNAs were synthesized on an automated DNA synthesizer using standard solid-phase phosphoramidite chemistry with 2′-*O*-pivaloyloxymethyl (2′-*O*-PivOM) nucleoside phosphoramidites as precursors to generate 2′OH after RNA deprotection [39] and 2′-*O*-acetylthiomethyl (2′-*O*-AcSM) G or C phosphoramidites as intermediates for the introduction of the linkers [40]. Elongations were carried out on a 1 µmol scale with a 180 s coupling step. At the end of the elongation process, reagents **1** and **2** were applied to the on-column RNA to perform a thiol-disulfide exchange reaction with the thiolate resulting from the acetyl cleavage of 2′-*O*-AcSM group with butylamine. The reactions afforded 2′-*O*-((4-((pyridin-2-yldisulfanyl)methyl)pentyl) disulfanyl) methyl 2′-*O*-PySS**R_1_**SSMe RNA bearing the pentyl spacer (R_1_) and the 2′-*O*-PySS**R_2_**SSMe RNA with the *p*-xylenyl spacer (R2; Scheme 4). Then, RNAs were fully deprotected and were released from solid-support upon an aqueous concentrated ammonia solution.

The 2′-*O*-functionalized RNAs were characterized by MALDI-TOF MS. The mass spectra showed the presence of three peaks, one corresponding to the expected mass (*m*/*z* 6944 for G_1_-R_1_ and C_11_-R_1_; *m*/*z* 6978 for G_1_-R_2_ and C_11_-R_2_) and the two others corresponding to the partial rupture of the disulfide bonds during laser irradiation resulting in mass losses of 109 Da and 243 Da for G_1_-R_1_ and C_11_-R_1_ and 109 Da and 277 Da for G_1_-R_2_ and C_11_-R_2_ (Figures S3–S6).

In the thiol-disulfide exchange reaction, a large excess of reagents **1** or **2** (500 equiv. per modification) in anhydrous BuNH_2_/THF (1/3) solution was required in order to limit the formation of dimeric RNA-RNA as side-products. Indeed, the proportion of desired RNAs significantly increased with the amount of reagents used. Thus with 20 or 100 equivalents of **2**, less than 10% of the desired products were obtained beside two major compounds that were identified by MALDI-TOF MS as RNA 2′-OH, due to an incomplete reaction, and RNA dimers resulting from disulfide bridge formation between two non-complementary RNA strands. With 500 equivalents of **2**, the peaks corresponding to expected RNAs represented about 30% of the overall peaks in the HPLC chromatograms. Further increase of the excess of reagents was not possible due to the low solubility of reagents at higher concentrations. 2′-*O*-PySS**R_1_**SSMe and 2′-*O*-PySS**R_2_**SSMe RNAs were obtained with high purity after purification by Ion-Exchange HPLC (IEX-HPLC; Figures S3–S7). Indeed, the *p*-xylenyl spacer (R_2_) allowed a better separation of the species by IEX-HPLC than the pentyl spacer (R_1_).

#### 2.1.3. Conjugation of Doxorubicin to 2′-O-PySSR_1_SSMe and 2′-O-PySSR_2_SSMe RNAs

**Scheme 5:**
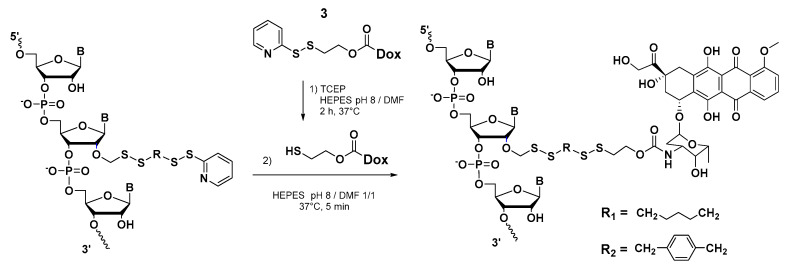
Synthesis of Dox-RNA conjugates in solution.

**Figure 1:**
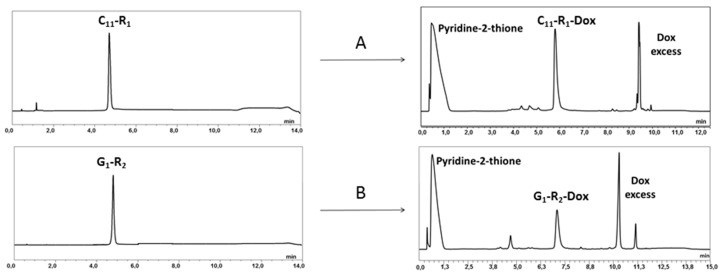
(**A**) Reverse-phase HPLC (RP-HPLC) profiles of pure RNA C_11_-R_1_ and of the crude mixture of C_11_-R_1_-Dox after conjugation with Dox-SS-Py derivative **3**. (**B**) RP-HPLC profiles of pure RNA G_1_-R_2_ and of the crude mixture of G_1_-R_2_-Dox after conjugation with Dox-SS-Py derivative **3**.

The five 2′-*O*-PySS**R**SSMe RNAs were conjugated to 2-(disulfanylpyridine)-ethylcarbamate doxorubicin (Dox-SS-Py) **3** [37] used in excess, through a two-step procedure adapted from the conjugation of RNA to diverse lipophilic compounds recently described by us [30] (Scheme 5). Dox-SS-Py **3** was first treated with tris(2-carboxyethyl)phosphine (TCEP) in HEPES buffer (pH = 8) and DMF for 2 h to give a thiol function. Then, this solution was directly added to RNAs G_1_-R_1_, C_11_-R_1_, G_1_-R_2_, C_11_-R_2_ upon stirring at 37 °C for 5 min and the reaction was followed by reverse-phase HPLC (RP-HPLC). In each chromatogram of the crude reaction mixture, three peaks were observed (Figure 1). The first peak eluting in the dead volume of the column was assigned to the pyridine-2-thione released during the reaction. One peak at a retention time around 10 min corresponds to the excess of Dox reagent. The third peak was assigned to the expected Dox**-**RNAs conjugates. Some minor peaks were also noticeable; they were probably due to the chemical degradation of RNA.

As demonstrated by the conjugation yields calculated from the peak areas of the conjugates in the HPLC chromatograms of the crude material (Table 2), the reactions were slightly more efficient with the pentyl spacer (91% G_1_-R_1_-Dox and 80% C_11_-R_1_-Dox) than with the *p*-xylenyl one (80% G_1_-R_2_-Dox and 69% C_11_-R_2_-Dox) in the linkers of the conjugates. This may indicate an influence of the flexibility of the linker on the conjugation conditions. Moreover, it appears that the conjugation at the 5′ end of the RNA was more efficient than the one in the middle of the sequence. Indeed, the conjugation of Dox to G_1_-R_1_ and G_1_-R_2_ proceeded with 91% and 80% yield, respectively in comparison with 80% and 69% obtained with C_11_-R_1_ and C_11_-R_2_, respectively. The accessibility of the nucleotide involved in the reaction may be responsible for this difference.

**Figure 2:**
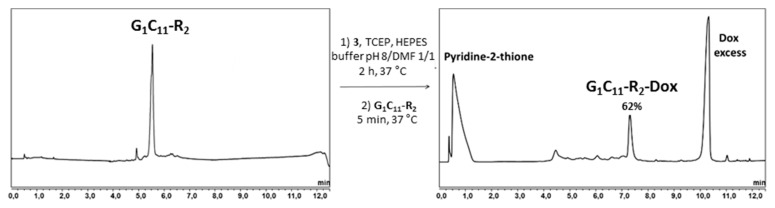
RP-HPLC profiles of solution phase conjugation of RNA **G_1_C_11_-R_2_** (left) with Dox-SS-Py derivative **3** to give bi-Dox-RNA conjugate **G_1_C_11_-R_2_-Dox** (right).

In order to evaluate multiple incorporations, we explored the conjugation reaction between Dox-SS-Py derivative **3** and RNA G_1_C_11_-R_2_ bearing two 2′-*O*-PySS**R_2_**SSMe modifications, on both 5′end (G_1_) and middle (C_11_) positions of the sequence. The same conditions were used as for the preparation of mono-Dox-RNA conjugates. After 5 min, as previously observed, RP-HPLC analysis of the crude product showed three major peaks with one peak corresponding to the desired RNA G_1_C_11_-R_2_-Dox (Figure 2). As expected, the conjugation yield (62%) and the recovery yield (26%) were slightly lower than yields obtained for mono-Dox-RNA conjugates nevertheless they were satisfactory enough to show that our method was efficient to ensure multi-conjugation of Dox to an RNA (Figure S12).

The crude Dox-RNAs were purified by IEX-HPLC and characterized by MALDI-TOF mass spectrometry (Figures S8–S12). It could be noted that the mass spectra of the mono-Dox-RNA conjugates systematically show two signals. One signal corresponds to the Dox-RNA conjugates (*m*/*z* 7482.8 for G_1_-R_1_-Dox and *m*/*z* 7484.0 for C_11_-R_1_-Dox, *m*/*z* 7516.5 for G_1_-R_2_-Dox and *m*/*z* 7515.3 for C_11_-R_2_-Dox) and the other signal at a lower mass (*m*/*z* 7085 for G_1_-R_1_-Dox and C_11_-R_1_-Dox, *m*/*z* 7117 for G_1_-R_2_-Dox and C_11_-R_2_-Dox), results from the rupture of the glycosidic bond of Dox during laser irradiation (loss of 398 Da). In the case of the bis-Dox-RNA conjugate G_1_C_11_-R_2_-Dox, two peaks of lesser mass were observed corresponding to the rupture of one and two glycosidic bonds of the Dox (Figure S12). Comparable recovery yields (between 39% and 49%) were determined for the mono-Dox-RNAs conjugates by UV absorbance of Dox at 481 nm (Table 2) [41]. The yield decrease between the conjugation yield and the yield of isolated products was assigned to losses during purification by IEX-HPLC and further desalting through the RP-cartridge.

### 2.2. Biophysical Properties of Dox-siRNA Conjugates

#### 2.2.1. Thermal Denaturation Studies of Dox-siRNAs

**Figure 3:**
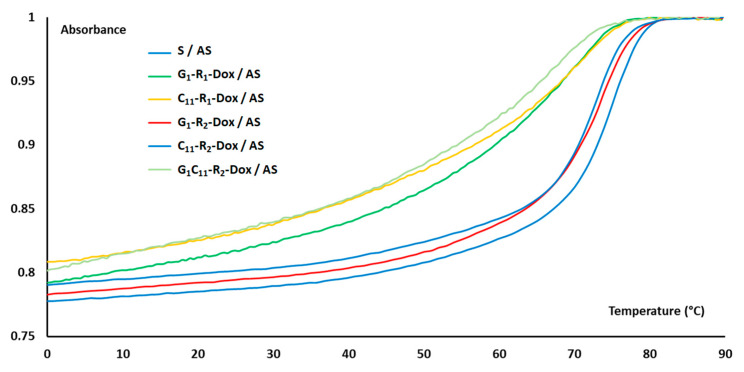
Melting curves of Dox-siRNA duplexes formed between Dox-RNA conjugates and their complementary unmodified RNA antisense (**AS**) strand.

The influence of the bound Dox on the thermal stability of the double-stranded RNA formed between Dox-RNA conjugates and their complementary unmodified RNA strand was evaluated by UV-melting experiments at 260 nm. All duplexes exhibited sigmoidal melting curves (Figure 3) with a single melting temperature (Table 2). Compared to the unmodified duplex S/AS (*T_m_* = 75.6 °C), all melting temperatures were decreased. However, this destabilization was not detrimental to the formation of regular RNA duplexes. Dox attached to RNA through the pentyl spacer R_1_ induced lower melting temperatures (Δ*T_m_* = −5.5 °C for G_1_-R_1_-Dox and −5.1 °C for C_11_-R_1_-Dox) than Dox linked with the *p*-xylenyl spacer R_2_ (Δ*T_m_* = −2.2 °C for G_1_-R_2_-Dox and −2.7 °C for C_11_-R_1_-Dox). The higher flexibility of the aliphatic spacer R_1_ might be responsible of enhanced freedom of Dox positioning, resulting in the stronger destabilization of the duplex. It is noteworthy that for one spacer, the effect was substantially the same regardless the position of Dox connection to siRNA. Surprisingly, for bi-Dox-siRNA conjugate G_1_C_11_-R_2_-Dox, the global destabilization (Δ*T_m_*/mod = −7.8 °C) was 1.6 fold higher than the additive effects of one R_2_-linked Dox in each duplex (Δ*T_m_* = −2.2 + −2.7 = −4.9 °C). Nevertheless, all Dox-siRNA conjugates form stable enough duplexes at physiological temperature to be used in biological assays.

#### 2.2.2. Circular Dichroism Studies of Dox-siRNAs

**Figure 4:**
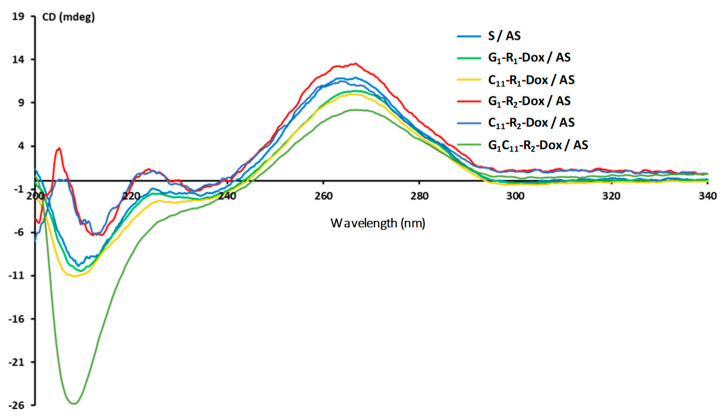
Circular dichroism (CD) spectroscopy curves of siRNAs duplexes formed between Dox-RNA conjugates with their complementary RNA AS strand (experiment performed at 41 °C).

Circular dichroism (CD) spectra of Dox-siRNAs exhibited the typical curve of A-form helix geometry, with a strong negative cotton effect around 210 nm and a positive cotton effect around 265 nm (Figure 4). Nevertheless, some variations in the intensity of these bands can be noticeable. In particular, a 40% decrease of the 265 nm band and a significant increase (more than 200%) of the negative band at 210 nm were observed for bis-Dox-siRNA conjugate G_1_C_11_-R_2_-Dox. This specific curve indicates a significant impact of two doxorubicin molecules on the double strand stacking confirming the thermal destabilization. Overall, all duplexes adopt an A-form helix required for RNA interference.

### 2.3. Stability of Dox-siRNAs in Human Serum

The siRNA duplexes were incubated in 10% human serum at 37 °C for 8 h and the enzymatic stabilities were monitored by polyacrylamide gel electrophoresis at several incubation times (Figure S13). Half-lives are reported in Table 2. The unmodified siRNA was rapidly degraded (t_1/2_ < 5 min) whereas a moderate increase of the enzymatic stability was noticed for mono-Dox-siRNAs with half-lives between 10 and 38 min. Neither the positioning of Dox in RNA sequence nor the structure of the linkers between Dox and RNA seems to impact the stability substantially. In contrast, the double conjugation of Dox to siRNA induced a further enhanced stability of the duplex (t_1/2_ = 48 min), indicating that multi-conjugation of Dox to both RNA strands might protect significantly the Dox-siRNA conjugates against enzymatic degradation.

### 2.4. Unmasking of a Dox-RNA Conjugate under Reductive Environment

**Figure 5:**
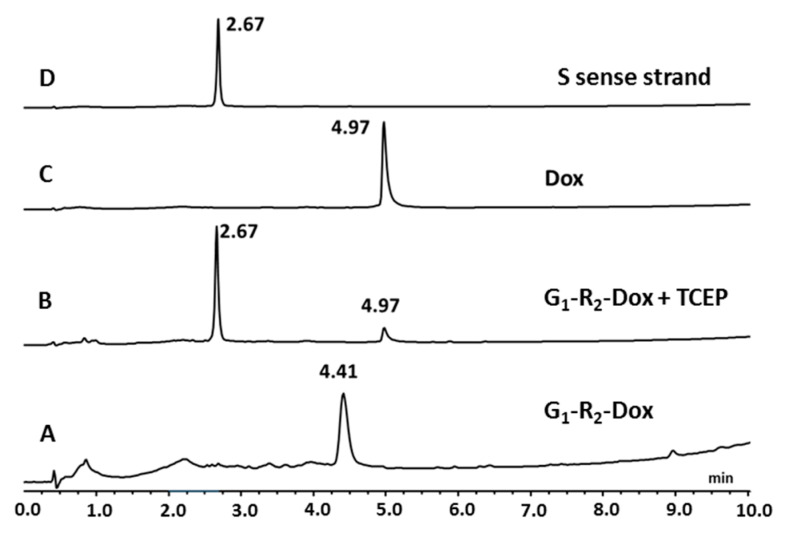
RP-HPLC profiles at 260 nm of **A**) G_1_-R_2_-Dox conjugate; **B**) crude mixture of G_1_-R_2_-Dox conjugate after 24 h incubation with TCEP at 37 °C; **C**) doxorubicin and **D**) RNA S sense strand of siEF-4.

To ascertain the self-immolative feature of the linkers between Dox and RNA, the release of the RNA (sense strand of siEF-4) and the free doxorubicin was evaluated in the presence of a reducing reagent. The G_1_-R_2_-Dox conjugate was incubated for 24 h with TCEP, and then analyzed by RP-HPLC (Figure 5). In parallel, free doxorubicin, unmodified RNA S and G_1_-R_2_-Dox conjugate without TCEP were analyzed in the same conditions by RP-HPLC. After 24 h, the peak corresponding to the starting G_1_-R_2_-Dox disappeared, indicating the effective cleavage of the disulfide bridges. The HPLC chromatogram displayed two new peaks, one at Rt 2.67 min was assigned to the unmodified RNA S, while the second peak at Rt 4.97 min was attributed to the native doxorubicin. As expected, this experiment demonstrates that the disulfide-based linker undergoes self-immolative fragmentation process under reductive conditions, releasing the intact bioactive drugs.

## 3. Materials and Methods

### 3.1. General Methods

MeOH was distilled over sodium. All other anhydrous solvents are commercial and used as is. All reactions were performed in anhydrous conditions under argon. Thin-layer chromatographies (TLC) were performed on silica plate 60 F254 Merck and the different spots were revealed at 254 nm. Column chromatography purifications were performed on silica gel (40–63 µm from Merck-Millipore). NMR experiments were accomplished on a Bruker 400 spectrometer at 20 °C. High Resolution Mass Spectrometry (HRMS) analyses were obtained with electrospray ionization (ESI) in the positive mode on a Quadrupole Time-of Flight (Q-TOF) MicroTof QII spectrometer (Bruker, Billerica, MA, USA).

Analytical and semi-preparative ion-exchange high performance liquid chromatography (IEX-HPLC) were performed on a Thermo Ultimate 3000 HPLC system (ThermoFisher Scientific, Waltham, MA, USA, Software Chromeleon 7.2) equipped with anion-exchange DNAPac PA 200 columns (4 mm × 250 mm for analysis or 9 mm × 250 mm for semi-preparative purposes, ThermoFisher Scientific), UV detection at 260 nm. The following HPLC solvent systems were used: 5% CH_3_CN in 25 mM Tris-HCl buffer, pH 8 (buffer A) and 5% CH_3_CN containing 400 mM NaClO_4_ in 25 mM Tris-HCl buffer, pH 8 (buffer B). Flow rates were 1.0 mL/min and 4 mL/min for analysis and semi-preparative purposes, respectively.

Analytical and semi-preparative reverse-phase high performance liquid chromatography (RP-HPLC) were performed on a Thermo Ultimate 3000 HPLC system equipped with a ThermoFisher Scientific LC Accucore™ column C_18_, 100 Å, 2.1 mm × 50 mm, Buffer A: 12.5 mM TEAAc in 1% CH_3_CN, Buffer B: 12.5 mM TEEAc in 80% CH_3_CN, 1 mL/min flow rate and UV detection at 260 nm).

MALDI-TOF mass spectra were recorded on an Axima assurance spectrometer equipped with a N_2_ laser (337 nm; Shimadzu, Kyoto, Japan) using a saturated solution of 2,4,6-trihydroxyacetophenone (THAP) as matrix in a mixture of acetonitrile/0.1 M ammonium citrate solution (1:1, *v*/*v*). Samples were mixed with the matrix in a (1:5, *v*/*v*) ratio, crystallized on a stainless steel plate and analyzed in negative mode. UV quantifications of RNAs were performed on a Varian Cary 300 Bio UV/visible spectrometer by measuring absorbance at 260 nm or 481 nm.

### 3.2. 1,5-bis(pyridin-2-yldisulfanyl)pentane 1

A solution of pentanedithiol (2.50 g, 18.4 mmol, 1.0 equiv) in dry MeOH (50 mL) was added dropwise under argon to a stirred solution of 2,2′-dithiodipyridine (12.0 g, 55.1 mmol, 3.00 equiv) in dry MeOH (200 mL). The yellow mixture was stirred at room temperature for 1.5 h to reach completion. The solvent was evaporated under vacuum. The crude material was purified by silica gel column chromatography with a step gradient of DCM and EtOAc (2–5%) followed, after evaporation of the solvent, by a RP column chromatography with a gradient of water and acetonitrile (0–60%). The desired compound **1** was obtained as colorless oil (2.01 g, 5.70 mmol, 31%). ^1^H-NMR (CDCl_3_, 400 MHz): δ(ppm) = 8.38 (ddd, *J* = 4.8 Hz, *J* = 1.8 Hz, *J* = 0.9 Hz, 2H, H_pyridyl_); 7.63 (dt, *J* = 8.0 Hz, *J* = 1.0 Hz, 2H, H_pyridyl_); 7.56 (td, *J* = 7.3 Hz, *J* = 1.7 Hz, 2H, H_pyridyl_); 7.00 (ddd, *J* = 7.3 Hz, *J* = 4.9 Hz, *J* = 1.2 Hz, 2H, H_pyridyl_); 2.70 (t, *J* = 7.2 Hz, 4H, 2CH_2_); 1.62 (p, *J* = 7.4 Hz, 4H, 2CH_2_); 1.47–1.41 (m, 2H, 1CH_2_). ^13^C-NMR (CDCl_3_, 100 MHz): 159.5 (2C_pyridyl_); 148.5 (2C_pyridyl_); 136.0 (2C_pyridyl_); 119.6 (2C_pyridyl_); 118.8 (2C_pyridyl_); 37.7 (2CH_2_); 27.4 (2CH_2_); 26.2 (CH_2_). HR-MS (ESI^+^): *m*/*z* calcd for C_15_H_19_N_2_S_4_ [M + H]^+^ 355.0431, Found 355.0434.

### 3.3. Bis(pyridin-2-yldisulfanyl)-p-xylene 2

A solution of 1,4-benzenedimethanethiol (2.50 g, 14.6 mmol, 1.00 equiv) in dry MeOH (50 mL) was added dropwise to a stirred solution of 2,2′-dithiodipyridine (9.70 g, 44 mmol, 3.00 equiv) in dry MeOH (200 mL) under argon. The yellow mixture was stirred at room temperature for 2.5 h to reach completion. The solvent was evaporated under vacuum. The crude material was purified by silica gel column chromatography with a step gradient of DCM and MeOH (0–10%). The desired compound **2** was obtained as pale yellow oil (2.1 g, 5.4 mmol, 37%). ^1^H-NMR (CDCl_3_, 400 MHz): δ(ppm)= 8.44 (d, *J* = 4.1 Hz, 2H, H_pyridyl_); 7.58–7.47 (m, 4H, H_pyridyl_); 7.19 (s, 4H, H_aro_); 7.05 (ddd, *J* = 7.0 Hz, *J* = 4.9 Hz, *J* = 1.1 Hz, 2H, H_aro_); 3.96 (s, 4H, 2CH_2_). ^13^C-NMR (CDCl_3_, 100 MHz): 158.9 (2C_pyridyl_); 148.2 (2C_pyridyl_); 136.1 (2C_aro_); 135.0 (2C_pyridyl_); 128.5 (4C_aro_); 119.7 (2C_pyridyl_); 118.8 (2C_pyridyl_); 42.4 (2CH_2_). HR-MS (ESI^+^): *m*/*z* calcd for C_18_H_17_N_2_S_4_ [M + H]^+^ 389.0275, Found 389.0274.

### 3.4. Solid-phase Synthesis of RNA Oligonucleotides

RNA oligonucleotides were synthesized using an ABI model 394 DNA/RNA synthesizer on a 1 µmol scale using 2′-*O*-PivOM and 2′-*O*-AcSM phosphoramidites (Chemgenes) and a long chain alkylamine (LCAA) controlled-pore glass (CPG) as solid support (Link technologies).

Oligonucleotides (ON) were assembled in TWIST^TM^ synthesis columns (Glen Research). Phosphoramidites were vacuum dried prior to their dissolution in extra dry acetonitrile (Biosolve) at 0.1 M concentration. Coupling for 180 s was performed with 5-benzylmercaptotetrazole (BMT, 0.3 M) as the activator. The oxidizing solution was 0.1 M iodine in THF/pyridine/H_2_O (78:20:2; *v*/*v*/*v*; Link Technologies). The capping step was performed for 160 s with a mixture of 5% phenoxyacetic anhydride (Pac_2_O) in THF and 10% *N*-methyl imidazole in THF (Link Technologies). Detritylation was performed with 3% TCA in CH_2_Cl_2_. After RNA assembly completion, the column was removed from the synthesizer and dried under a stream of argon.

### 3.5. Synthesis of Modified 2′-O- PySSRSSMe RNAs G_1_-R_1_, C_11_-R_1_, G_1_-R_2_, C_11_-R_2_ and G_1_C_11_-R_2_

After RNA elongation, the solid support was treated with 2 mL of a 0.4 M solution of **1** or **2** (500 equiv.) and a 2.5 M butylamine (0.5 mL) solution in anhydrous THF (1.5 mL). The solution was applied to the synthesis column using two glass syringes filled with 4Å molecular sieves (5 beads each), and pushed back and forth through the synthesis column for 15 min. Then the solution was removed and the solid-support was washed with anhydrous THF followed by a 1 min flush with argon. Finally, the solid support was treated with a 30% aqueous ammonia solution for 3 h at 30 °C. The solution was evaporated in the presence of isopropylamine (13% of total volume) under reduced pressure and redissolved in water (1.5 mL) before analysis and purification.

The crude oligonucleotides were analyzed by IEX-HPLC and MALDI-TOF spectrometry then purified by semi-preparative IEX-HPLC. The pure fractions of each ON were pooled in a 100 mL round-bottomed flask and were concentrated to dryness under reduced pressure. The residues were dissolved in 100 mL TEAAc buffer, pH 7 and desalted using a C_18_ cartridge (Sep-Pak^®^, Waters) equilibrated with a 100 mM TEAAc buffer solution. The desired compounds were eluted with a 12.5 mM TEAAc/CH_3_CN (1:1, *v*/*v*) solution in a 50 mL round-bottomed flask and were lyophilized. The residues were dissolved in 1.5 mL water (divided in 3 portions 0.8 mL, 0.4 mL and 0.3 mL for flask rinsing), transferred to a 2 mL Eppendorf-vial and lyophilized once again. Purified RNAs were characterized by MALDI-TOF MS and quantified by UV measurement.

### 3.6. Synthesis of Dox-RNA Conjugates G_1_-R_1_-Dox, C_11_-R_1_-Dox, G_1_-R_2_-Dox, C_11_-R_2_-Dox and G_1_C_11_-R_2_-Dox

A 10 mM solution of 2-(disulfanylpyridine)-ethylcarbamate doxorubicin **3** was prepared in DMF. The solution (100 equiv) was diluted with an equal volume of HEPES buffer (7.5 mM HEPES, 34 mM NaCl, 1 mM MgCl_2_ and 0.05 mM EDTA, pH 8) and a solution of TCEP (50 nmol/µL; 80 equiv). The mixture was stirred for 2 h at 37 °C using a thermoshaker (Thermal Shake lite ^®^). Then the solution was added to the freeze-dried RNAs G_1_-R_1_, C_11_-R_1_, G_1_-R_2_, C_11_-R_2_ and G_1_C_11_-R_2_ in a 0.5 mL Eppendorf tube and stirred at 37 °C for 5 min using a thermoshaker (Thermal Shake lite^®^). The reaction was stopped by addition of a large excess of HEPES buffer pH 5. The Dox-RNA conjugates were immediately purified by RP-HPLC, characterized by MALDI-TOF MS using the procedures described above.

### 3.7. Thermal Denaturation Studies of Dox-siRNA Duplexes

T_m_ experiments were performed using a CARY 300 UV Spectrophotometer (Varian Inc., Palo Alto, CA, USA) equipped with a Peltier temperature controller and thermal analysis software. The samples were prepared by mixing ON solutions of RNA sense and antisense strands together to give 1.5 µM final concentration in 1 mL of 10 mM sodium cacodylate buffer, 100 mM NaCl, pH 7 in a 1 cm path length quartz cell. A heating–cooling–heating cycle in the 0–90 °C temperature range with a gradient of 0.5 °C min^-1^ was applied. T_m_ values were determined from the maxima of the first derivative plots of absorbance recorded at 260 nm versus temperature. The T_m_ values from two independent experiments were accurate within ± 0.5 °C.

### 3.8. CD Spectroscopy Studies of Dox-siRNA Duplexes

CD spectra were recorded on a Jasco J-815 spectropolarimeter (Jasco, Easton, MD, USA). The samples were prepared by mixing solutions of RNA sense and antisense strands together to give 1.5 µM final concentration in 1 mL of 10 mM sodium cacodylate buffer, 100 mM NaCl, pH 7 in a 1 cm path length quartz cell. Measurements were performed at 1 °C with the wavelength range set to 200–340 nm and a scanning speed of 100 nm.min^−1^. Raw data were acquired over 2 scans.

### 3.9. Serum Stability of Dox-siRNA Duplexes

For annealing complementary RNA strands, equimolar amounts of sense and antisense strands were incubated in H_2_O MilliQ for 5 min at 90 °C followed by slow cooling to room temperature. RNA duplexes (C = 100 µM, V = 4 µL) were incubated in a mixture of phosphate buffered saline solution (PBS) 10 × (2.4 µL), H_2_O MilliQ (15.2 µL) and human serum AB type solution (Sigma; 2.4 µL) at 37 °C. After 1 min, 5 min, 15 min, 30 min, 60 min, 120 min, 240 min and 480 min, 3 µL were withdrawn and immediately frozen in liquid nitrogen and stored at −80 °C until analysis. Each sample was then analyzed by gel electrophoresis (15% polyacrylamide) 20 W, 1.5 h at 4 °C. The gels were stained with GelRed 3 × (VWR) in a 0.1 M NaCl solution for 1 h and revealed with an UV transilluminator. Band analysis was performed using ImageJ Software (Broken Symmetry Software V:1.4.3.67). Half-life lives were determined by the hypothesis of a 1 first-order kinetic and the calculation were performed on Microsoft Office Excel with an exponential trend line given by y(t) = Ae^-kt^ (0.90 ≤ R^2^ ≤ 1.00).

### 3.10. Unmasking of G_1_-R_2_-Dox Conjugate in the Presence of TCEP

Of the conjugate G_1_-R_2_-Dox (10 µL) 1 nmol was mixed with 90 µL of 7.5 mM HEPES buffer, 34 mM NaCl, 1 mM MgCl_2_, 0.05 mM EDTA, pH 8 and 5 µL of a 50 mM solution of TCEP in the same HEPES buffer. The mixture was incubated at 37 °C. After 24 h, the sample was analyzed by RP-HPLC. RNA references S (sense strand) and G_1_-R_2_-Dox were prepared in water. Doxorubicin and modified doxorubicin **3** as references were dissolved in DMF and diluted with buffer before RP-HPLC analyses. The solution of **3** was also treated for 5 min at 37 °C with a large excess of a 50 mM solution de TCEP to ensure the thiol formation before HPLC analysis.

## 4. Conclusions

In summary, the synthesis of five Dox-RNA conjugates in a siRNA-doxorubicin co-delivery strategy was reported. The covalent linkage between RNA and the anticancer drug was performed through disulfide-based self-immolative linkers, aiming to release both active compounds in reducing conditions. These extensively used SILs are known to take benefit from the selective activation upon disulfide cleavage in the reducing environment in the cells. Two disulfide SILs, one with a pentane spacer and the other with a *p*-xylene spacer between two disulfide bonds have been introduced to connect RNA to Dox. The conjugation was performed via two thiol-disulfide exchange reactions. The first one was achieved on-column at 2′-*O*-AcSM ribonucleotide of RNA to anchor the linker and the second one consisted in linking in solution a thiol-modified doxorubicin derivative to RNAs at different positions, affording the Dox-RNA conjugates with satisfactory yields. Multi-conjugation was also validated by the synthesis of a bis-Dox-RNA conjugate.

These Dox-RNA conjugates were used to design five Dox-siRNAs. They displayed a moderate increase of stability in human serum and a sufficient thermal duplex stability under physiological conditions for RNA interference activity. The self-immolative feature of the linkers was confirmed by the recovery of both native RNA and doxorubicin under reductive conditions. Biological evaluation of the Dox-siRNA conjugates remains to be further explored. Thus, these reduction-responsive siRNA-drug bioconjugates might represent an alternative approach in the development of co-delivery strategies to optimize chemotherapy efficacy by siRNA silencing to overcome multi-drug resistance of cancer cells.

## Figures and Tables

**Table 1:** Sequence, mass spectrometry data and yields of 2′-*O*-PySS**R_1_**SSMe and 2′-*O*-PySS**R_2_**SSMe RNAs prepared in this work.

RNA	Sequence 5′ → 3′ ^1^	MALDI-TOF MS ^2^		Conjugation Yield (%) ^3^
		Calcd.	Found	
S	GCA GCA GAA CCC UUC UUA UGA	/	/	/
G_1_-R_1_	G_R1_CA GCA GAA CCC UUC UUA UGA	6945.6	6944.4	32
C_11_-R_1_	GCA GCA GAA CC_R1_UUC UUA UGA	6945.6	6944.1	28
G_1_-R_2_	G_R2_CA GCA GAA CCC UUC UUA UGA	6979.6	6978.1	24
C_11_-R_2_	GCA GCA GAA CC_R2_C UUC UUA UGA	6979.6	6978.6	35
G_1_C_11_-R_2_	G_R2_CA GCA GAA CC_R2_C UUC UUA UGA	7296.2	7294.3	15

^1^ N_R_ = 2′-*O*-PySSRSSMe ribonucleoside; R_1_ for pentyl and R_2_ for *p*-xylenyl. ^2^ negative mode. ^3^ percentage of peak area corresponding to the desired RNAs in the crude material as calculated by integration of the IEX-HPLC chromatogram monitored at 260 nm.

**Table 2:** Data on Dox-RNA conjugates.

RNA	Sequence 5′ → 3′ ^1^	MALDI-TOF MS ^2^		Conjugation Yield (%) ^3^	Recovery Yield (%) ^4^	*T*_m_^5^ (°C)	Half-life HS (min) ^6^
		Calcd.	Found				
S G_1_-R_1_-Dox	GCAGCAGAACCCUUCUUAUGA G_Dox_CAGCAGAACCCUUCUUAUGA	/ 7482.1	/ 7482.8	/ 91	/ 39	75.6 70.1	<5.0 13
C_11_-R_1_-Dox	GCAGCAGAACC_Dox_CUUCUUAUGA	7482.1	7484.0	80	46	70.5	38
G_1_-R_2_-Dox	G_Dox_CAGCAGAACCCUUCUUAUGA	7517.1	7516.5	80	49	73.4	20
C_11_-R_2_-Dox	GCAGCAGAACC_Dox_CUUCUUAUGA	7517.1	7515.3	69	43	72.9	10
G_1_C_11_-R_2_-Dox	G_Dox_CAGCAGAACC_Dox_CUUCUUAUGA	8375.1	8375.3	62	26	67.8	48

^1^ N_Dox_ = Dox-ribonucleotide conjugates. ^2^ negative mode. ^3^ percentage of peak area corresponding to the Dox-RNA conjugate in the crude material as calculated by integration of the RP-HPLC chromatogram monitored at 260 nm. ^4^ Recovery yield based on UV absorption at 481 nm (λmax Dox). ^5^ *T*_m_ values (°C) obtained from UV melting curves at 260 nm with 1.5 µM strand concentration in 10 mM sodium cacodylate, 100 mM NaCl, pH 7.0. Data are averages of two hybridization/melting cycles. Estimated errors in *T*_m_ = ± 0.5 °C. Complementary strand AS: ^5^^′^AUA AGA AGG GUU CUG CUG CCC^3′^. ^6^ half-lives were determined in 10% human serum (HS).

## Supplementary Materials

The following are available online at https://www.mdpi.com/1420-3049/25/11/2714/s1, Figure S1: 400 MHz ^1^H-NMR and 100 MHz ^13^C-NMR spectra (CDCl_3_) of 1, Figure S2: 400 MHz ^1^H-NMR and 100 MHz ^13^C-NMR spectra (CDCl_3_) of 2, Figure S3: IEX-HPLC and MALDI-TOF MS analyses of purified RNA G_1_-R_1_, Figure S4: IEX-HPLC and MALDI-TOF MS analyses of purified RNA C_11_-R_1_, Figure S5: IEX-HPLC and MALDI-TOF MS analyses of purified RNA G_1_-R_2_, Figure S6: IEX-HPLC and MALDI-TOF MS analyses of purified RNA C_11_-R_2_, Figure S7: IEX-HPLC and MALDI-TOF MS analyses of purified RNA G_1_C_11_-R_2_, Figure S8: A: HPLC profile of the conjugation reaction of RNA G_1_-R_1_ to Dox-SS-Py 3, B: IEX-HPLC and MALDI-TOF MS analyses of purified G_1_-R_1_-Dox conjugate, Figure S9: IEX-HPLC and MALDI-TOF MS analyses of purified C_11_-R_1_-Dox, Figure S10: IEX-HPLC and MALDI-TOF MS analyses of purified G_1_-R_2_-Dox conjugate, Figure S11: A: HPLC profile of solution phase conjugation of C_11_-R_2_ with Dox-SS-Py derivative 3, B: IEX-HPLC and MALDI-TOF MS analyses of purified C_11_-R_2_-Dox, Figure S12: IEX-HPLC and MALDI-TOF MS analyses of purified G_1_C_11_-R_2_-Dox conjugate, Figure S13: Gel electrophoresis of Dox-siRNA conjugates incubated in 10% human serum AB type over an 8 h period at 37 °C.
