# Supplementary material for: Conjugation of Doxorubicin to siRNA Through Disulfide-based Self-immolative Linkers
Source: Molecules. 2020 Jun 11;25(11):2714. doi: 10.3390/molecules25112714 (PMC7321315; doi:10.3390/molecules25112714)
Supplement: Supplementary file 1 [file molecules-25-02714-s001.pdf]

Supplementary Information

Conjugation of doxorubicin to siRNA through disulfide-based self-immolative linkers

Florian Gauthier [1], Jean-Rémi Bertrand[2], Jean-Jacques Vasseur [1,*], Christelle Dupouy [1,*] and Françoise Debart [1]

[1] IBMM, University of Montpellier, CNRS, ENSCM, Montpellier, France
[2] UMR 8203 CNRS, Université Paris-Sud, Gustave Roussy, University Paris-Saclay, Villejuif Cedex, France
* Correspondence: jean-jacques.vasseur@umontpellier.fr; christelle.dupouy@umontpellier.fr

^{1}H-NMR and ^{13}C-NMR spectra 2
Figure S1: 400 MHz ^{1}H-NMR and 100 MHz ^{13}C-NMR spectra ($CDCl_3$) of 1 2
Figure S2: 400 MHz ^{1}H-NMR and 100 MHz ^{13}C-NMR spectra ($CDCl_3$) of 2 3
HPLC chromatograms & MALDI-TOF spectra of purified oligonucleotides 4
Figure S3: IEX-HPLC and MALDI-TOF MS analyses of purified RNA G_1-R_1 4
Figure S4: IEX-HPLC and MALDI-TOF MS analyses of purified RNA C_{11}-R_1 5
Figure S5: IEX-HPLC and MALDI-TOF MS analyses of purified RNA G_1-R_2 6
Figure S6: IEX-HPLC and MALDI-TOF MS analyses of purified RNA C_{11}-R_2 7
Figure S7: IEX-HPLC and MALDI-TOF MS analyses of purified RNA G_1C_{11}-R_2 8
Figure S8: 9
A: HPLC profile of the conjugation reaction of RNA G_1-R_1 to Dox-SS-Py 3. 9
B: IEX-HPLC and MALDI-TOF MS analyses of purified G_1-R_1-Dox conjugate. 9
Figure S9: IEX-HPLC and MALDI-TOF MS analyses of purified C_{11}-R_1-Dox 10
Figure S10: IEX-HPLC and MALDI-TOF MS analyses of purified G_1-R_2-Dox conjugate 11
Figure S11: 12
A: HPLC profile of solution phase conjugation of C_{11}-R_2 with Dox-SS-Py derivative 3. ...12
B: IEX-HPLC and MALDI-TOF MS analyses of purified C_{11}-R_2-Dox 12
Figure S12: IEX-HPLC and MALDI-TOF MS analyses of purified G_1C_{11}-R_2-Dox conjugate 13
Enzymatic Stability of Dox-siRNAs in human serum 14
Figure S13: Gel electrophoresis of Dox-siRNA conjugates incubated in 10% human serum AB type over an 8 h period at 37 °C. 14

^{1}H-NMR and ^{13}C-NMR spectra

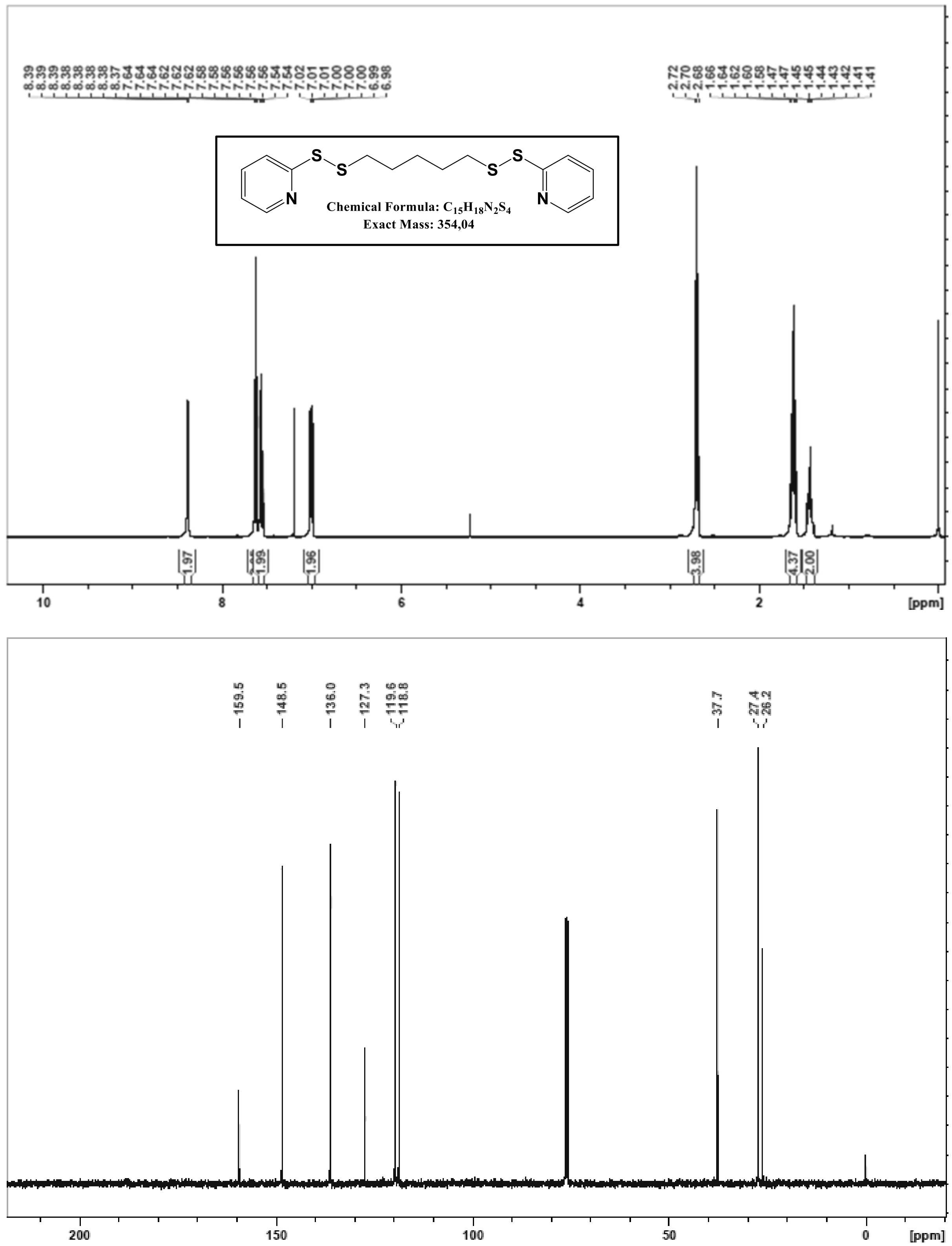

Figure 1. 400 MHz ^{1}H-NMR and 100 MHz ^{13}C-NMR spectra ($CDCl_3$) of **1.**

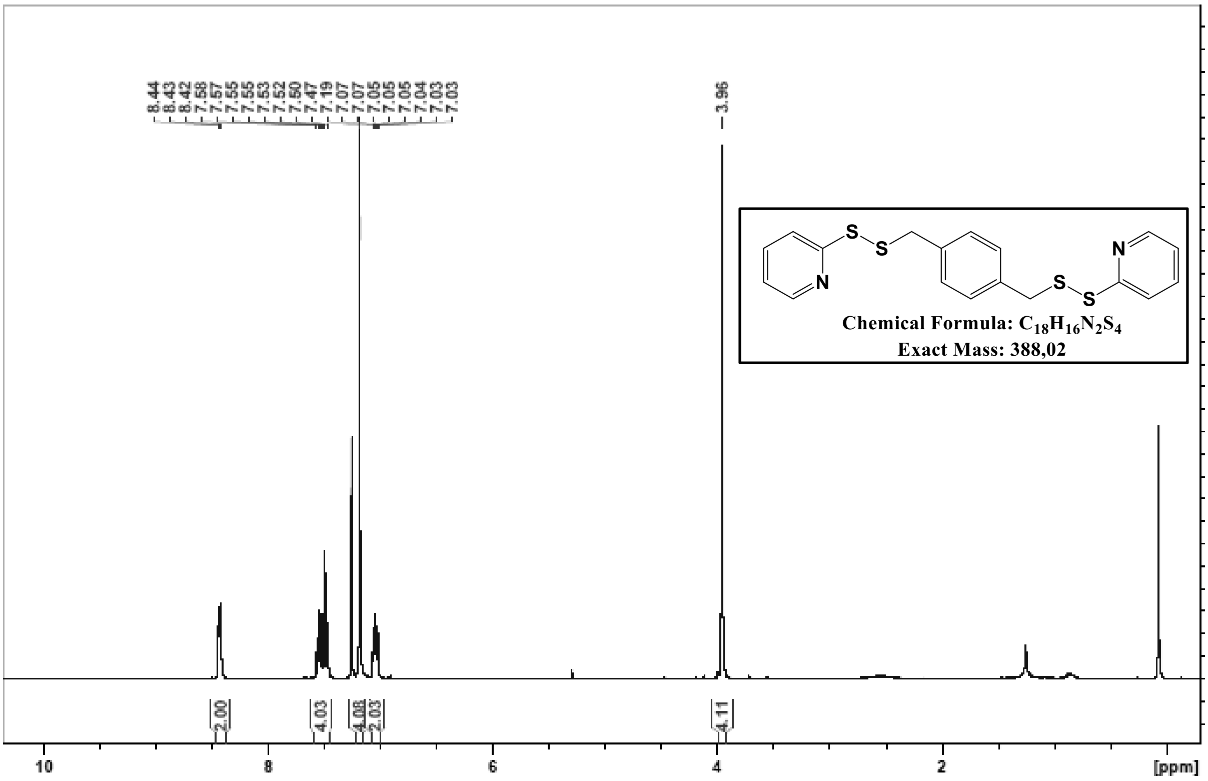

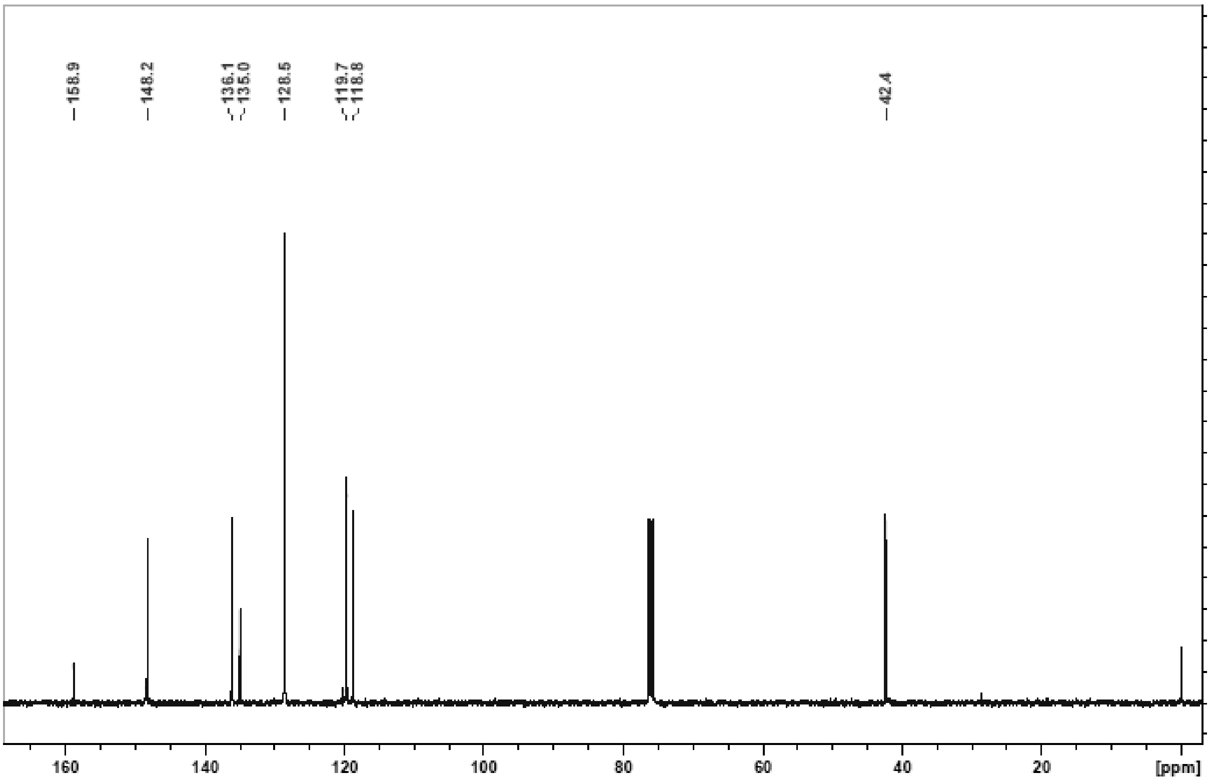

Figure S2: 400 MHz ^{1}H-NMR and 100 MHz ^{13}C-NMR spectra ($CDCl_3$) of **2**

HPLC chromatograms & MALDI-TOF spectra of purified oligonucleotides

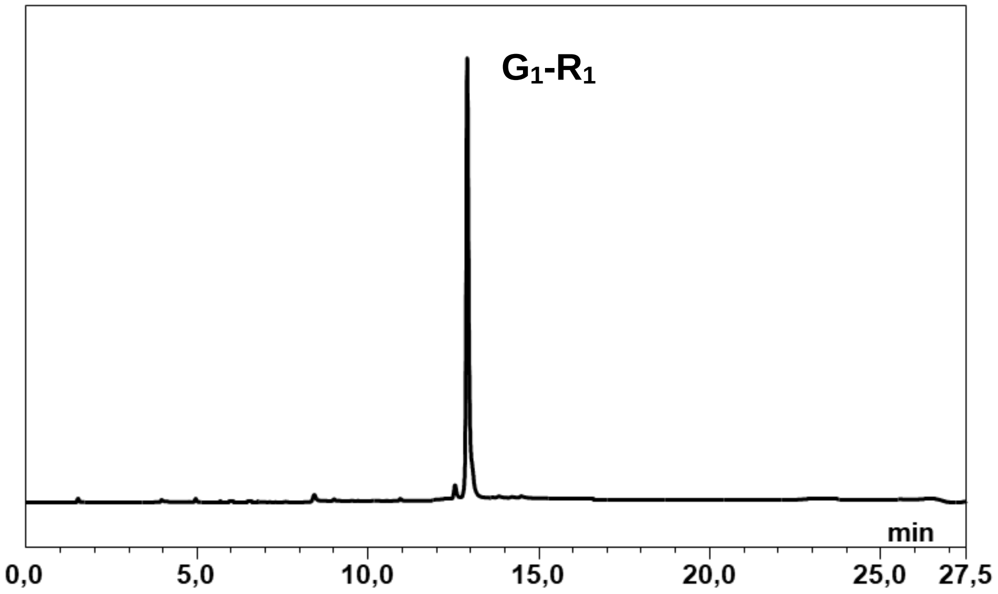

IEX-HPLC analysis conditions: DNAPac® PA100, 4 x 250 mm, elution with a 20 min linear gradient of 0 to 75% of B in eluent A. Column temperature 30 °C. Flow rate 1.5 mL.min^{-1}. λ 260 nm.

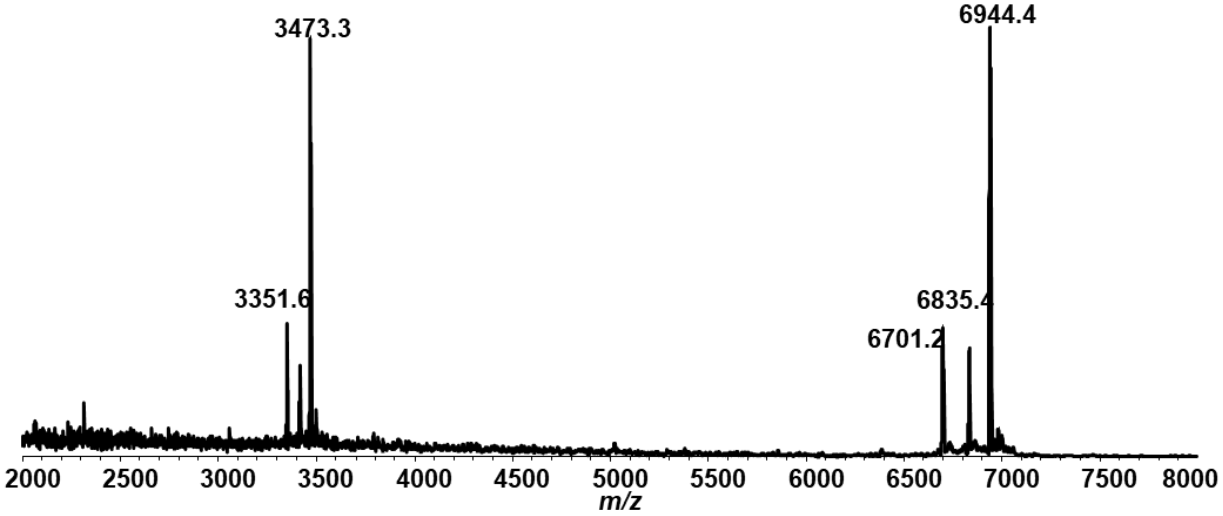

Figure S3: IEX-HPLC and MALDI-TOF MS analyses of purified RNA **G_1-R_1**

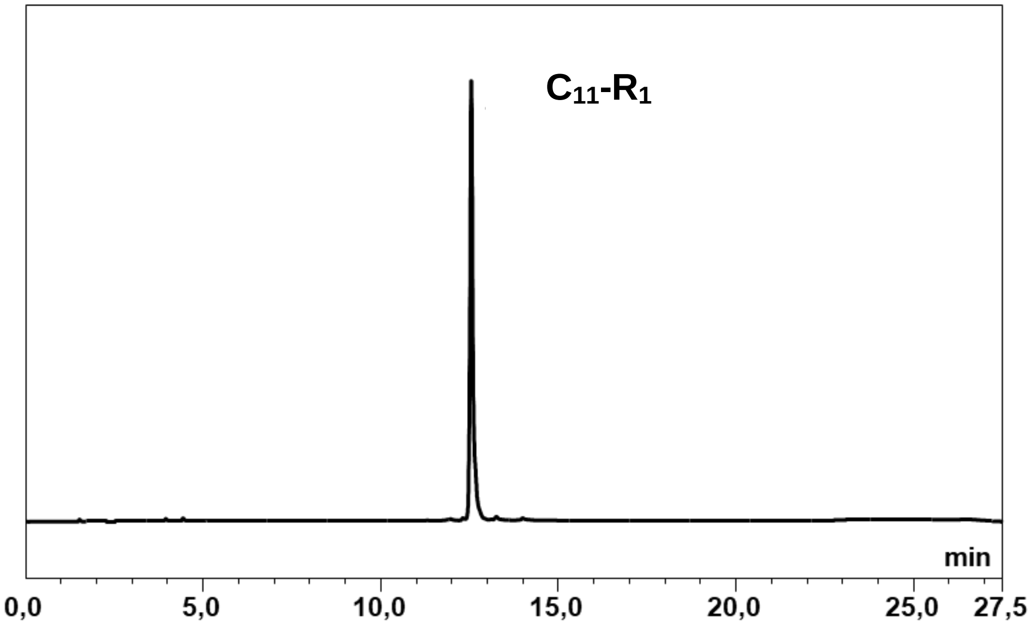

IEX-HPLC analysis conditions: DNAPac® PA100, 4 x 250 mm, elution with a 20 min linear gradient of 0 to 75% of B in eluent A. Column temperature 30 °C. Flow rate 1.0 mL.min^{-1}. λ 260 nm.

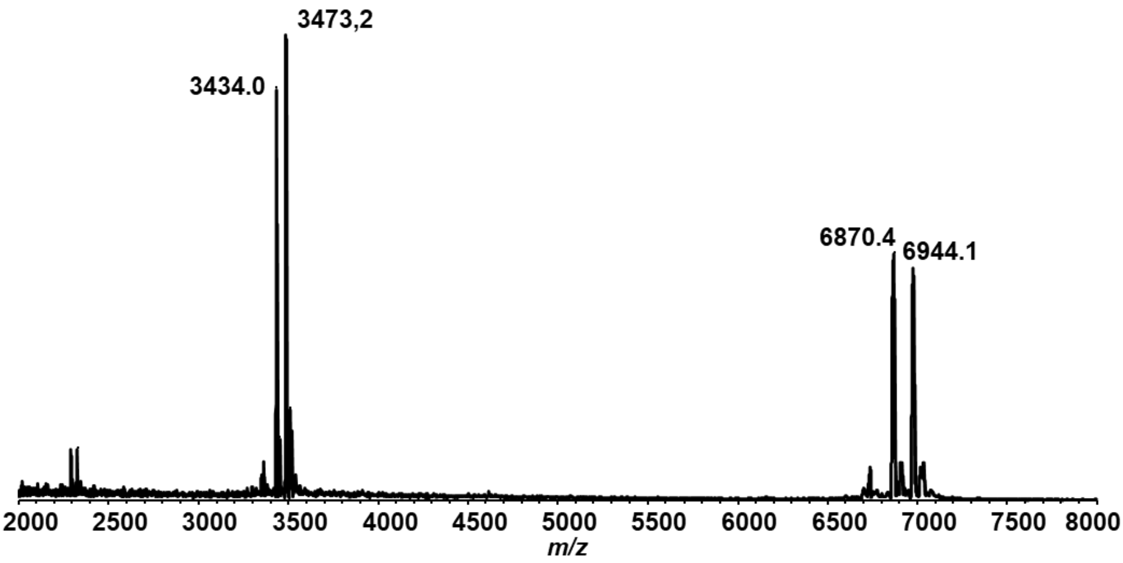

Figure S4: IEX-HPLC and MALDI-TOF MS analyses of purified RNA **C_{11}-R_1**

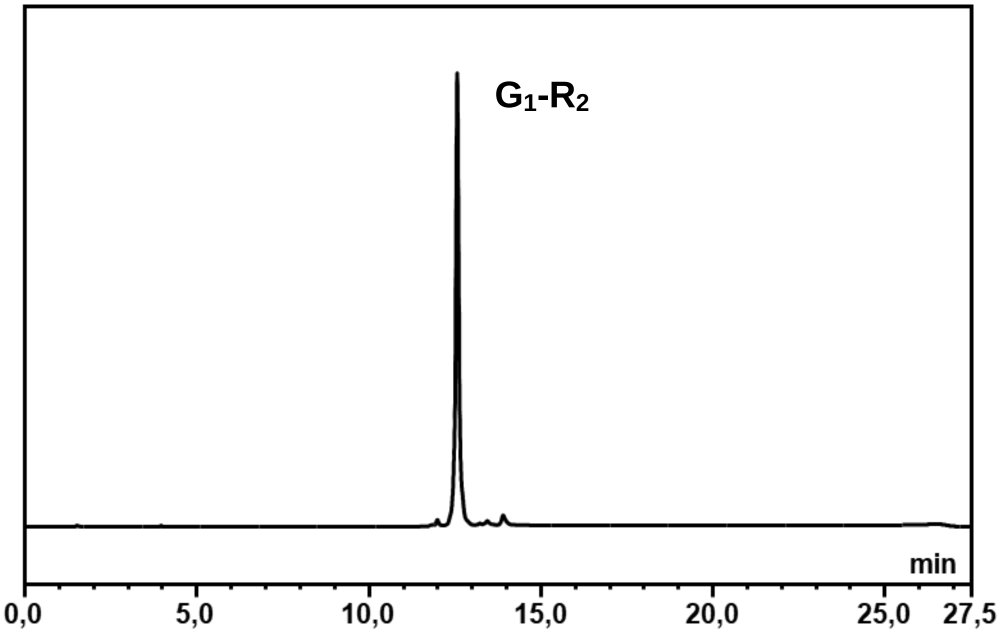

IEX-HPLC analysis conditions: DNAPac® PA100, 4 x 250 mm, elution with a 20 min linear gradient of 10 to 90% of B in eluent A. Column temperature 30 °C. Flow rate 1.0 mL.min^{-1}. λ 260 nm.

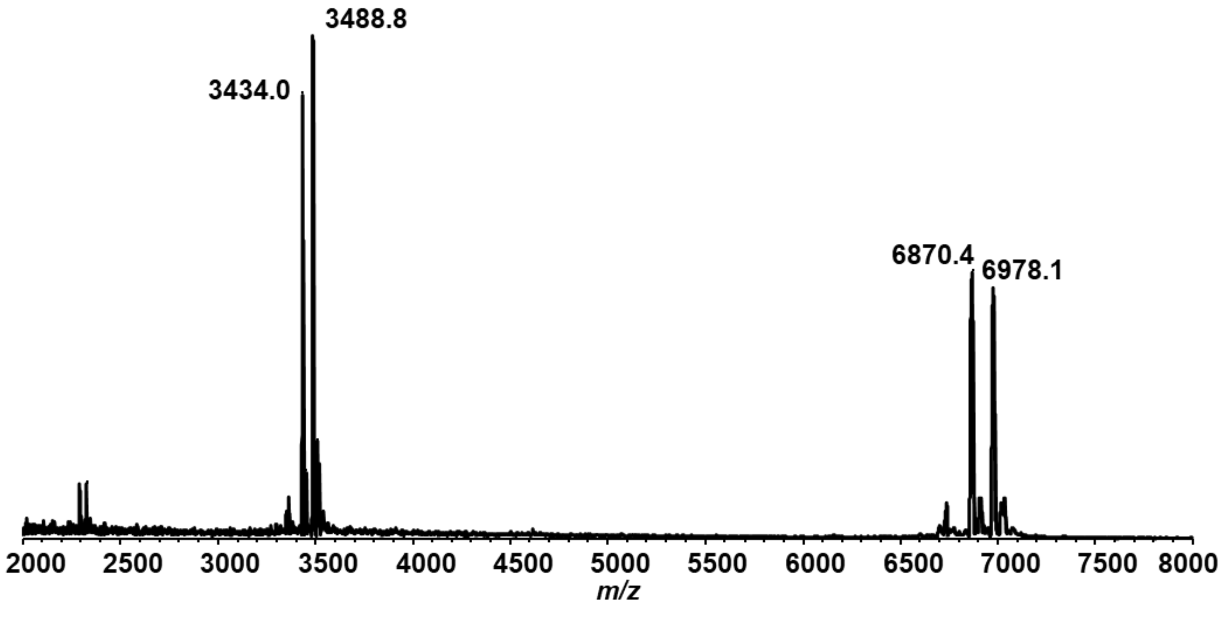

Figure S5: IEX-HPLC and MALDI-TOF MS analyses of purified RNA $\mathbf{G_1}$-$\mathbf{R_2}$

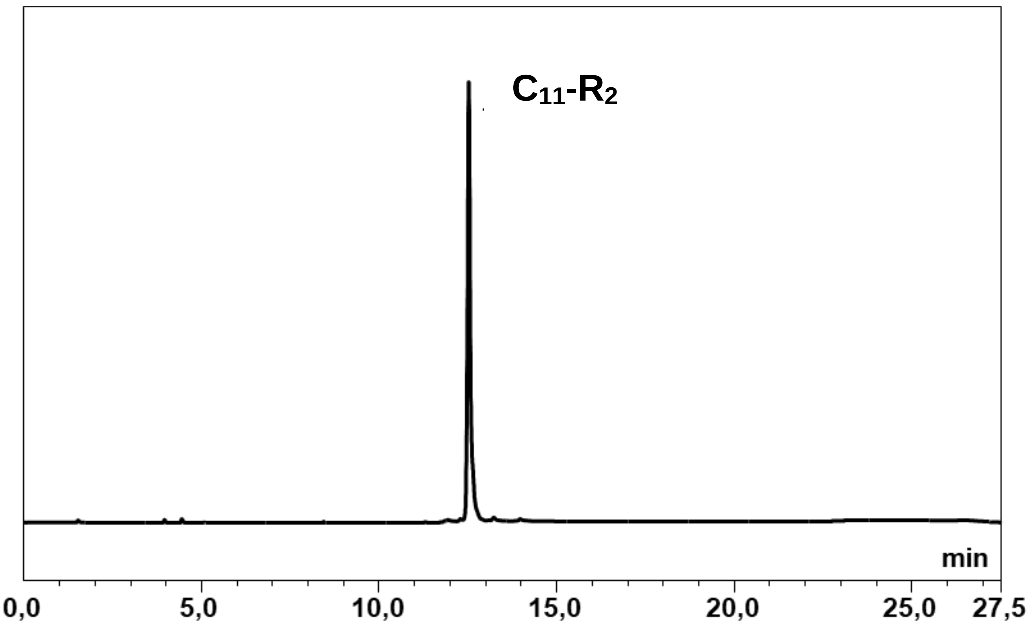

IEX-HPLC analysis conditions: DNAPac® PA100, 4 x 250 mm, elution with a 20 min linear gradient of 30 to 100% of B in eluent A. Column temperature 30 °C. Flow rate 1.0 mL.min^{-1}*. λ 260 nm.*

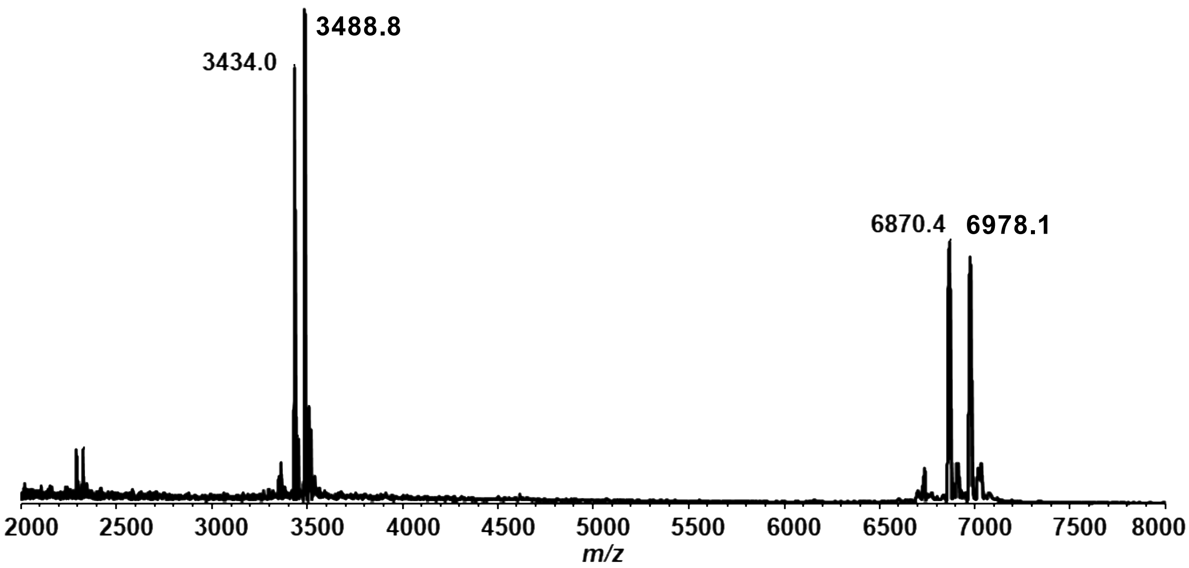

Figure S6: IEX-HPLC and MALDI-TOF MS analyses of purified RNA $\mathbf{C_{11}}$**-**$\mathbf{R_2}$

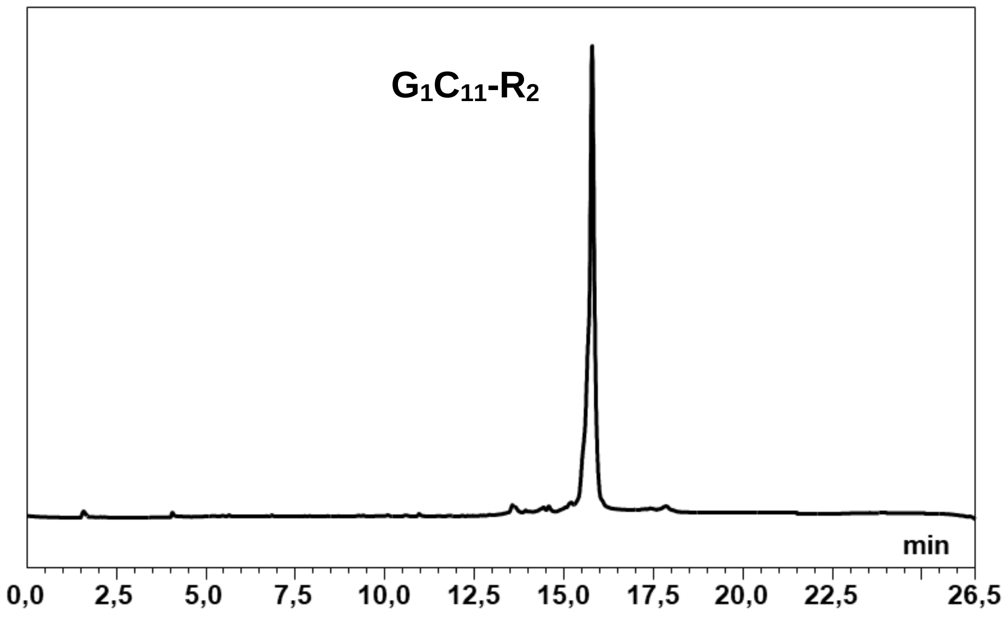

IEX-HPLC analysis conditions: DNAPac® PA100, 4 x 250 mm, elution with a 20 min linear gradient of 40 to 100% of B in eluent A. Column temperature 75 °C. Flow rate 1.0 mL.min^{-1}. λ 260 nm.

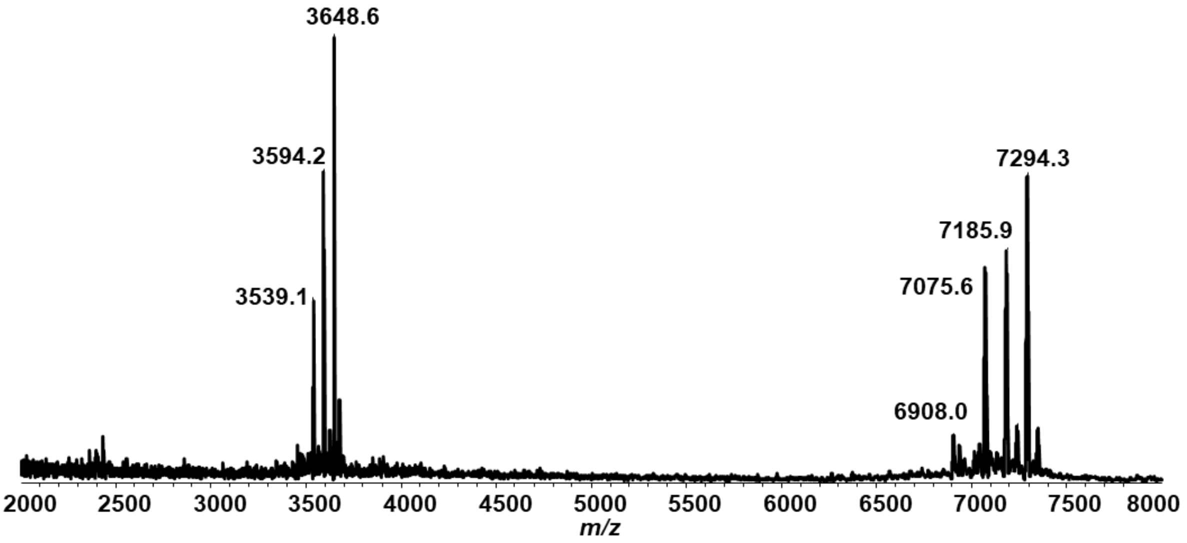

Figure S7: IEX-HPLC and MALDI-TOF MS analyses of purified RNA **G_1C_{11}-R_2**

A

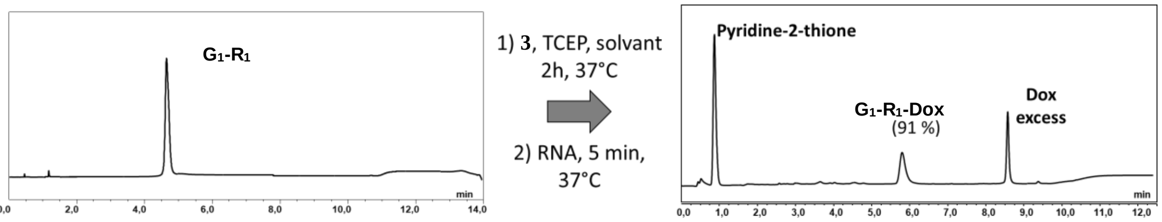

RP-HPLC chromatograms of RNA $\mathbf{G_1}$**-**$\mathbf{R_1}$ *(left), and of the crude conjugation mixture to* **Dox** *(right). Conditions: ThermoFisher Scientific LC Accucore® C18, 100Å, 2.1 x 50 mm, elution with a 10 min linear gradient of 0% to 50% of B (12.5 mM TEAAc in 1% ACN) in eluent A (12.5 mM TEAAc in 80% ACN). Column temperature 30°C. Flow rate 1.0 mL.min*$^{-1}$*. UV monitoring at 260 nm (percentage of peak area corresponding to the Dox-RNA conjugate in RNA crude material).*

B

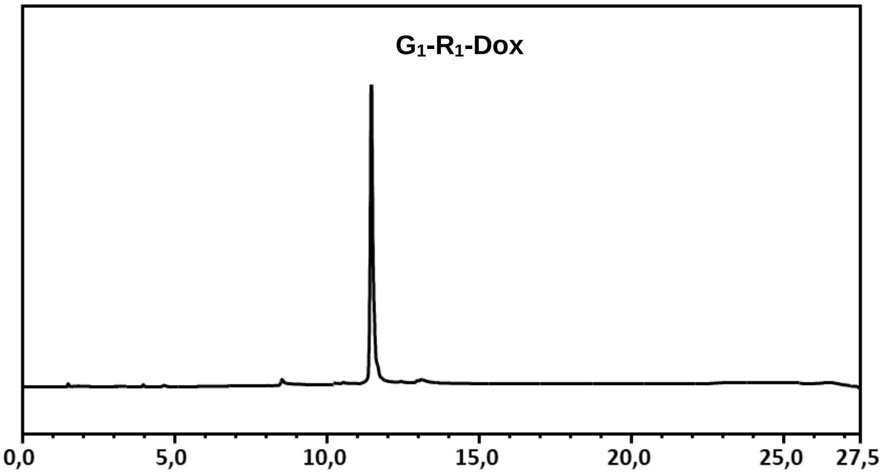

IEX-HPLC analysis conditions: column DNAPac® PA100, 4 x 250 mm, elution with a 20 min linear gradient of 30 to 100% of B in eluent A. Column temperature 75 °C. Flow rate 1.0 mL.min^{-1}*. λ 260 nm.*

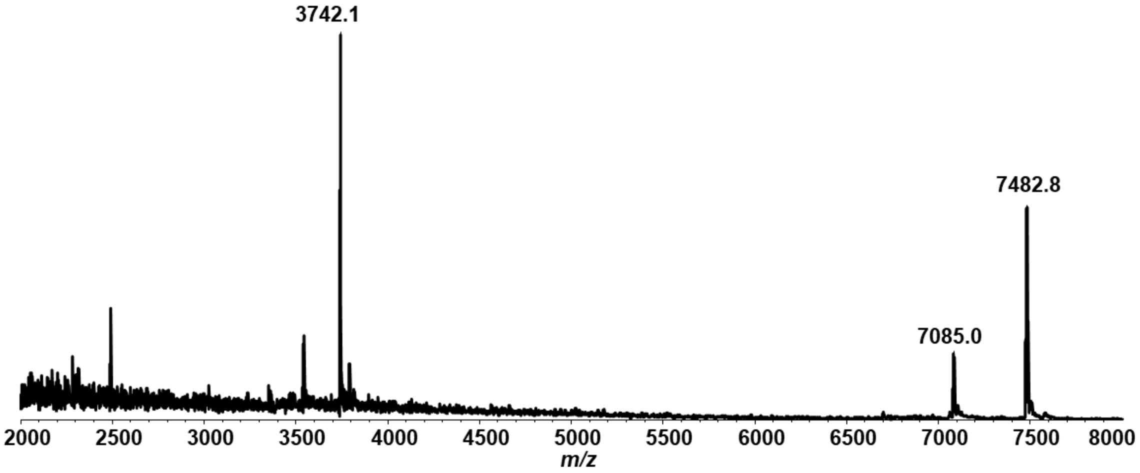

Figure S8:

A: HPLC profile of the conjugation reaction of RNA $\mathbf{G_1}$**-**$\mathbf{R_1}$ to Dox-SS-Py **3**.

B: IEX-HPLC and MALDI-TOF MS analyses of purified $\mathbf{G_1}$**-**$\mathbf{R_1}$**-Dox** conjugate.

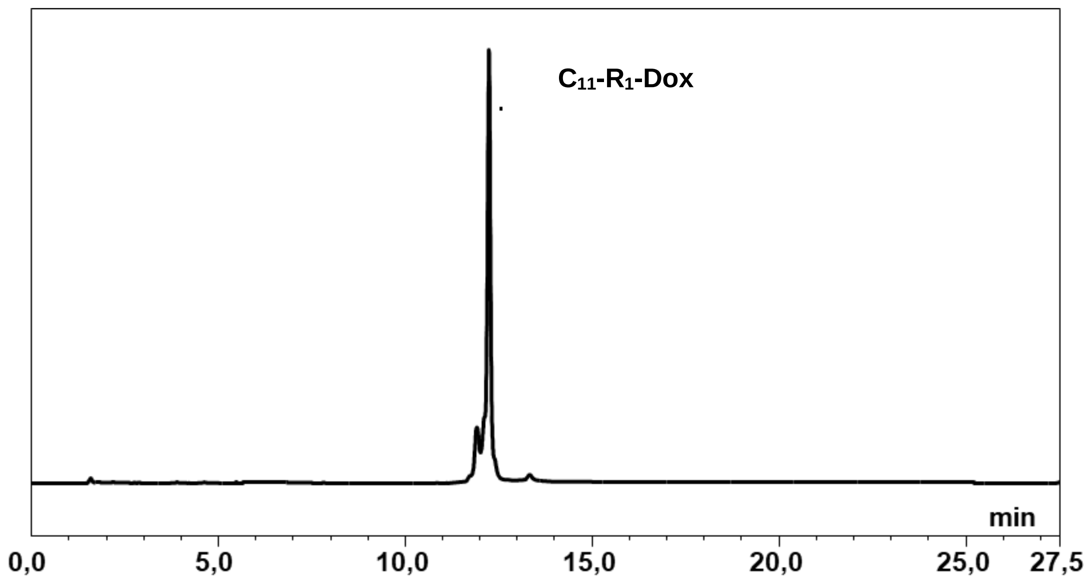

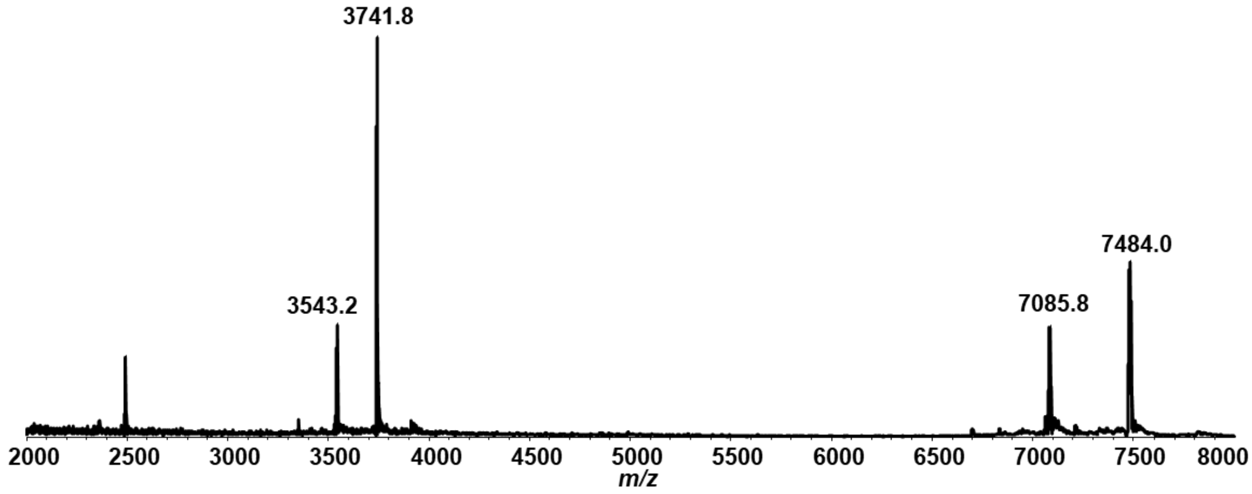

Figure S9: IEX-HPLC and MALDI-TOF MS analyses of purified **C_{11}-R_1-Dox**

IEX-HPLC analysis conditions: DNAPac® PA100, 4 x 250 mm, elution with a 20 min linear gradient of 40 to 100% of B in eluent A. Column temperature 75 °C. Flow rate 1.5 mL.min^{-1}. λ 260 nm.

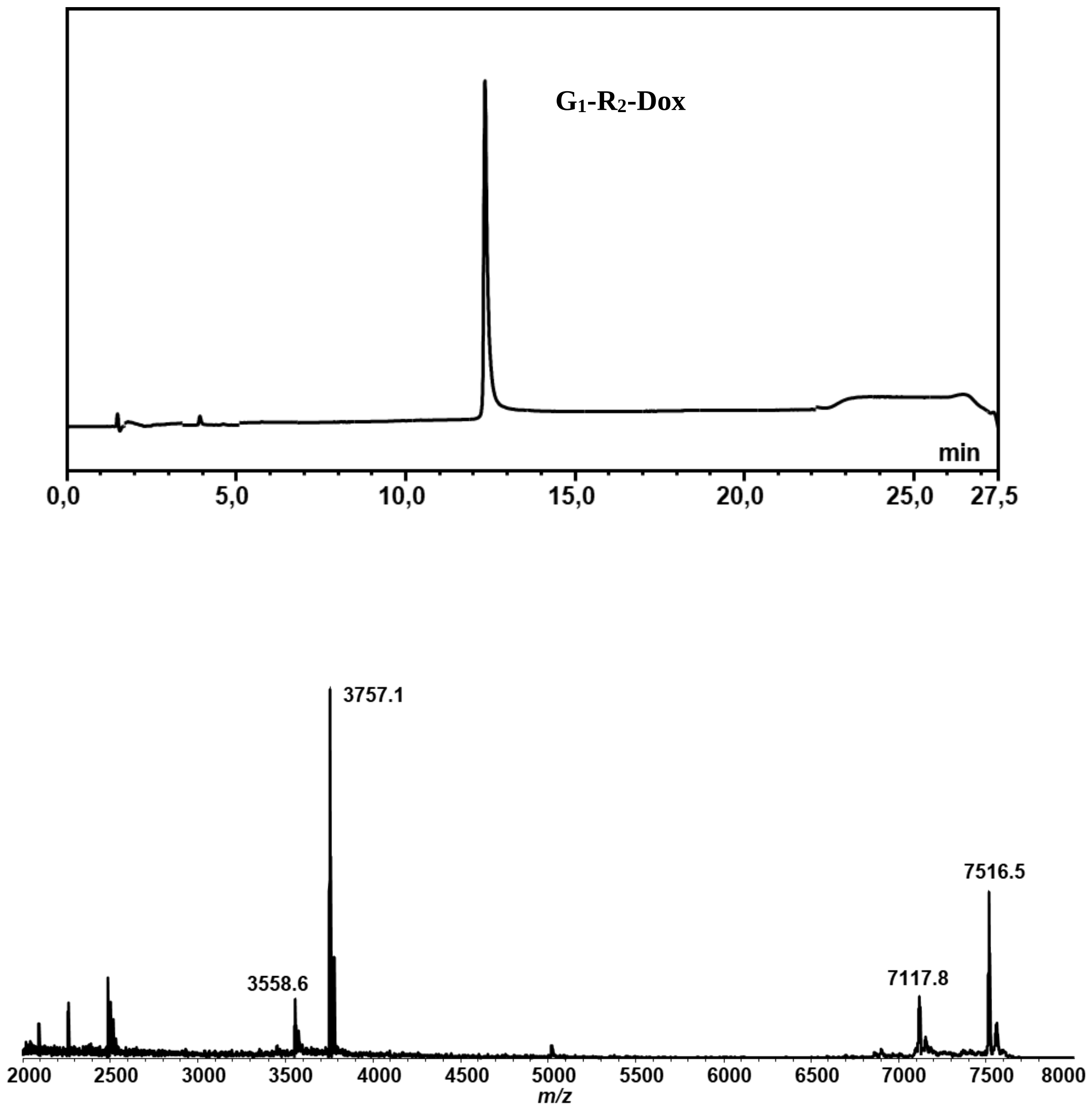

Figure S10: IEX-HPLC and MALDI-TOF MS analyses of purified G_1-R_2-Dox conjugate

IEX-HPLC analysis conditions: DNAPac® PA100, 4 x 250 mm, elution with a 20 min linear gradient of 30 to 90% of B in eluent A. Column temperature 75 °C. Flow rate 1.0 mL.min^{-1}. λ 260 nm.

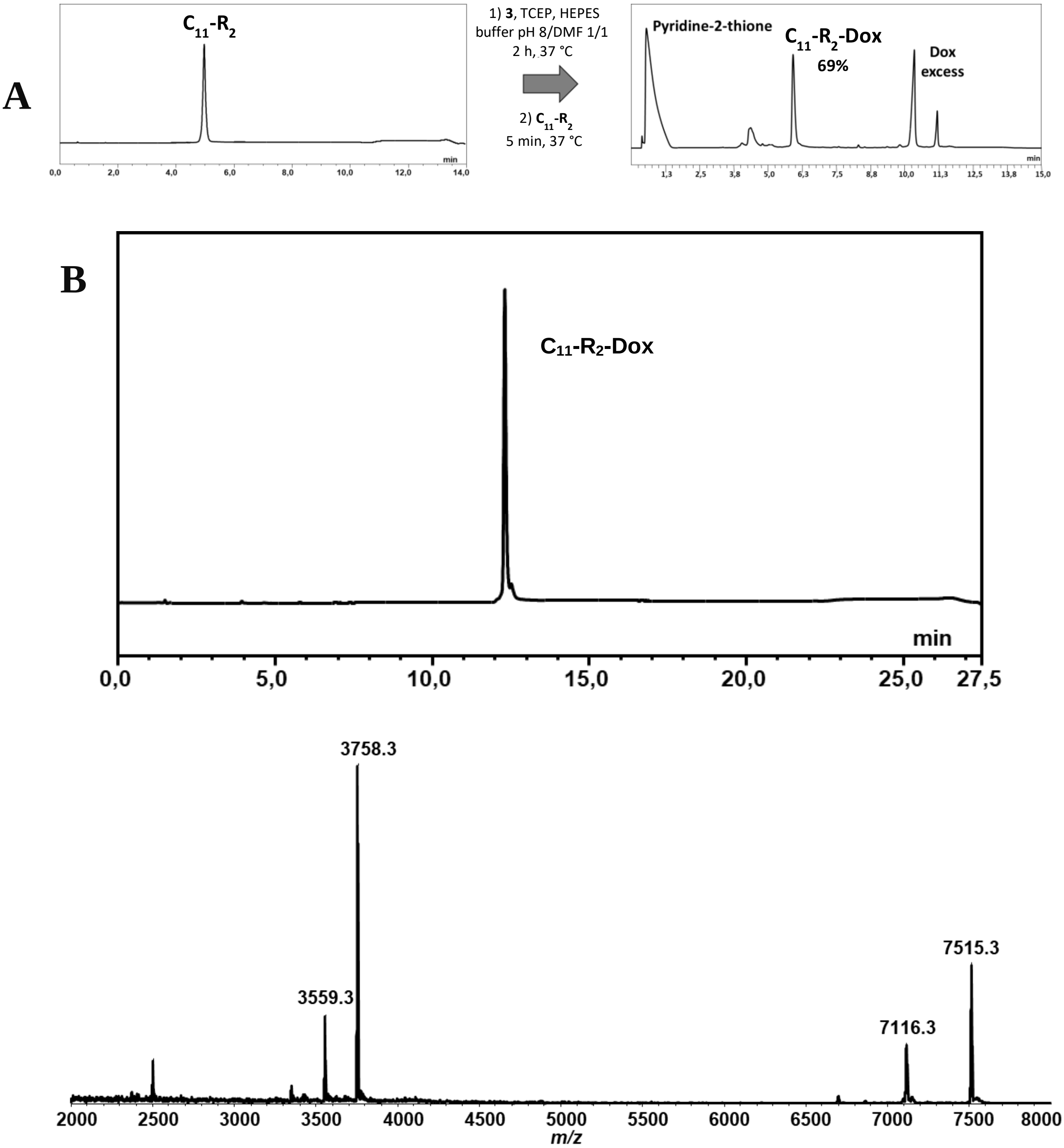

Figure S11:

A: HPLC profile of solution phase conjugation of $\mathbf{C_{11}\text{-}R_2}$ with Dox-SS-Py derivative **3**.

RP-chromatogram of $\mathbf{C_{11}\text{-}R_2}$ *(left), and of crude conjugation mixture* $\mathbf{C_{11}\text{-}R_2\text{-}Dox}$ *(right). Conditions: ThermoFisher Scientific LC Accucore® C18, 100Å, 2.1 x 50 mm, elution with a 10 min linear gradient of 0% to 50% of B (12.5 mM TEAAc in 1% ACN) in eluent A (12.5 mM TEAAc in 80% ACN). Column temperature 30°C. Flow rate 1.0 mL.min*$^{-1}$*. UV monitoring at 260 nm (percentage of peak area corresponding to the Dox-RNA conjugate in RNA crude material)*

B: IEX-HPLC and MALDI-TOF MS analyses of purified $\mathbf{C_{11}\text{-}R_2\text{-}Dox}$

IEX-HPLC analysis conditions: DNAPac® PA100, 4 x 250 mm, elution with a 20 min linear gradient of 30 to 100% of B in eluent A. Column temperature 75 °C. Flow rate 1.0 mL.min^{-1}*. λ 260 nm.*

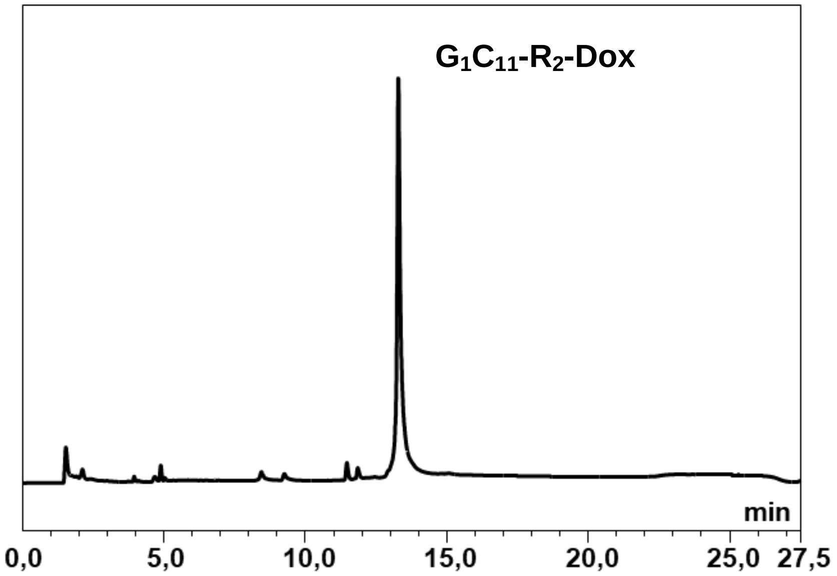

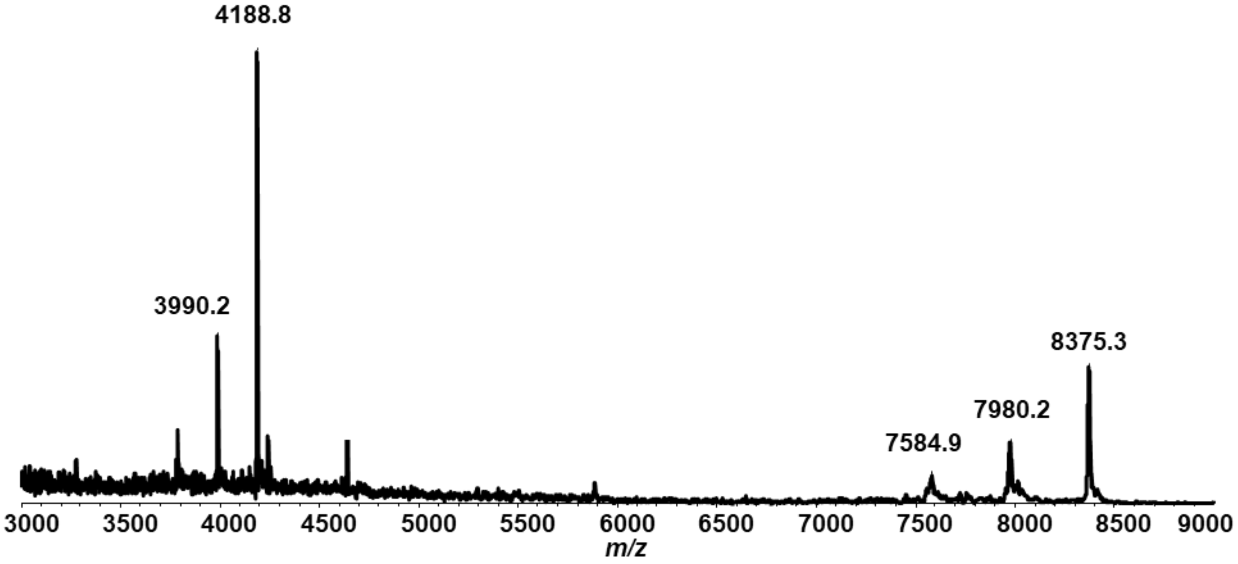

Figure S12: IEX-HPLC and MALDI-TOF MS analyses of purified **G_1C_{11}-R_2-Dox** conjugate

IEX-HPLC analysis conditions: DNAPac® PA100, 4 x 250 mm, elution with a 20 min linear gradient of 40 to 100% of B in eluent A. Column temperature 75 °C. Flow rate 1.0 mL.min^{-1}. λ 260 nm.

Enzymatic Stability of Dox-siRNAs in human serum

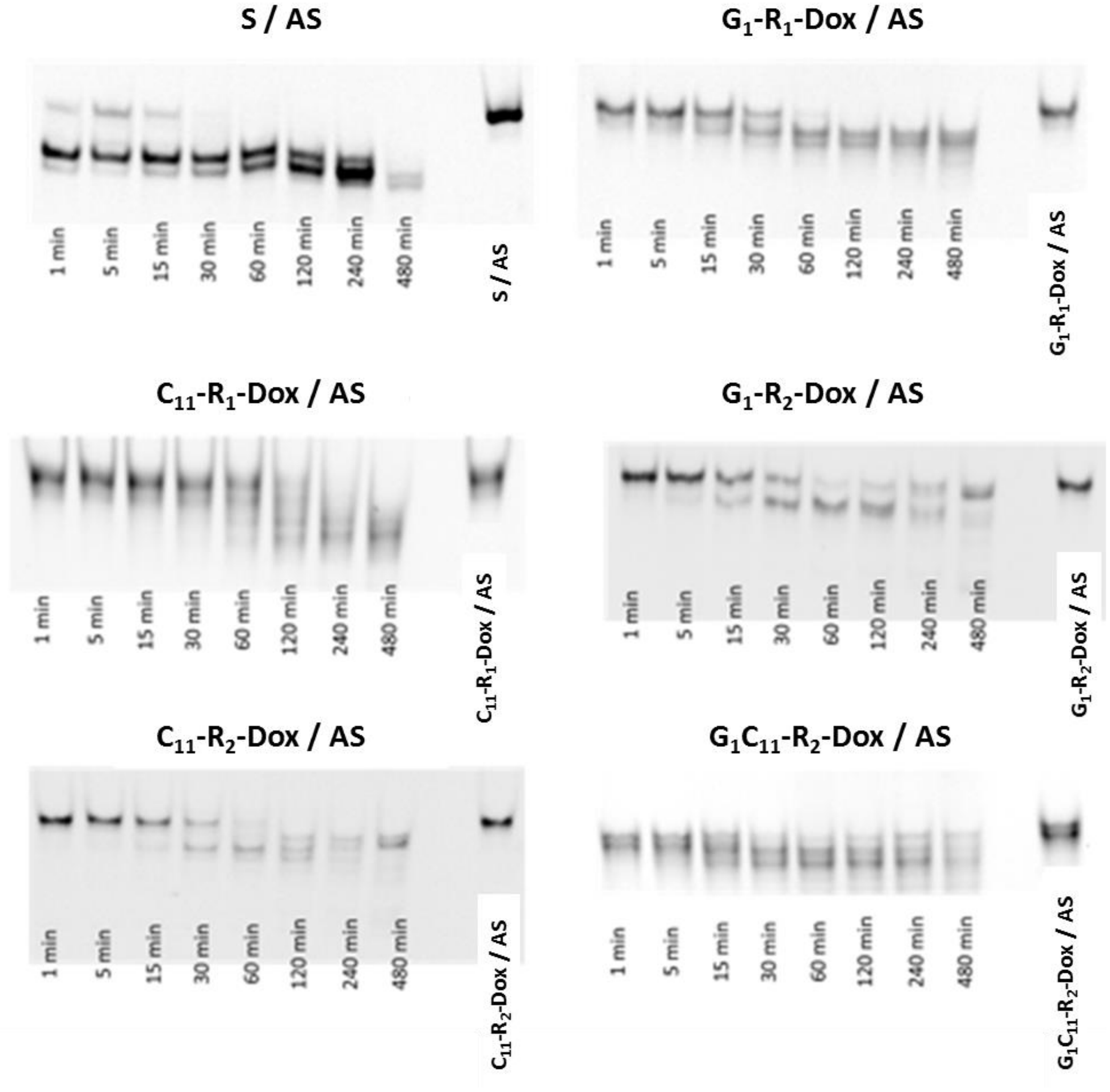

Figure S13: Gel electrophoresis of Dox-siRNA conjugates incubated in 10% human serum AB type over an 8 h period at 37 °C.
